# Modeling of the Nitric Oxide Transport in the Human Lungs

**DOI:** 10.3389/fphys.2016.00255

**Published:** 2016-06-28

**Authors:** Cyril Karamaoun, Alain Van Muylem, Benoît Haut

**Affiliations:** ^1^Ecole polytechnique de Bruxelles, Transfers, Interfaces and Processes, Université libre de BruxellesBrussels, Belgium; ^2^Chest Department, Erasme University Hospital and Université libre de BruxellesBrussels, Belgium

**Keywords:** nitric oxide, lungs, model, asthma, transport

## Abstract

In the human lungs, nitric oxide (NO) acts as a bronchodilatator, by relaxing the bronchial smooth muscles and is closely linked to the inflammatory status of the lungs, owing to its antimicrobial activity. Furthermore, the molar fraction of NO in the exhaled air has been shown to be higher for asthmatic patients than for healthy patients. Multiple models have been developed in order to characterize the NO dynamics in the lungs, owing to their complex structure. Indeed, direct measurements in the lungs are difficult and, therefore, these models are valuable tools to interpret experimental data. In this work, a new model of the NO transport in the human lungs is proposed. It belongs to the family of the morphological models and is based on the morphometric model of Weibel (1963). When compared to models published previously, its main new features are the layered representation of the wall of the airways and the possibility to simulate the influence of bronchoconstriction (BC) and of the presence of mucus on the NO transport in lungs. The model is based on a geometrical description of the lungs, at rest and during a respiratory cycle, coupled with transport equations, written in the layers composing an airway wall and in the lumen of the airways. First, it is checked that the model is able to reproduce experimental information available in the literature. Second, the model is used to discuss some features of the NO transport in healthy and unhealthy lungs. The simulation results are analyzed, especially when BC has occurred in the lungs. For instance, it is shown that BC can have a significant influence on the NO transport in the tissues composing an airway wall. It is also shown that the relation between BC and the molar fraction of NO in the exhaled air is complex. Indeed, BC might lead to an increase or to a decrease of this molar fraction, depending on the extent of the BC and on the possible presence of mucus. This should be confirmed experimentally and might provide an interesting way to characterize the extent of BC in unhealthy patients.

## 1. Introduction

For more than 20 years now, nitric oxide (NO) has been shown to be of a striking importance in various physiological processes. This ubiquitous molecule contributes, among other roles, to vasodilatation (Palmer et al., 1987) and neurotransmission (Bredt and Snyder, 1989). In the human lungs, NO acts as a bronchodilatator, by relaxing the bronchial smooth muscles. It is also closely linked to the inflammatory status of the lungs, owing to its antimicrobial activity (Moncada et al., 1991). Furthermore, the molar fraction of NO in the exhaled air (the so-called FE_NO_) has been shown to be higher for asthmatic patients than for healthy patients (Kharitonov et al., 1994). Recent studies managed to link the FE_NO_ and the diagnosis of the disease, as well as its management, in selected cases of asthma (Barnes et al., 2010; Haccuria et al., 2014). However, although the measurement of the FE_NO_ tends to become a standard during an asthma consultation, it is still difficult to link this measurement with a precise description of the pathology.

**Figure 1:**
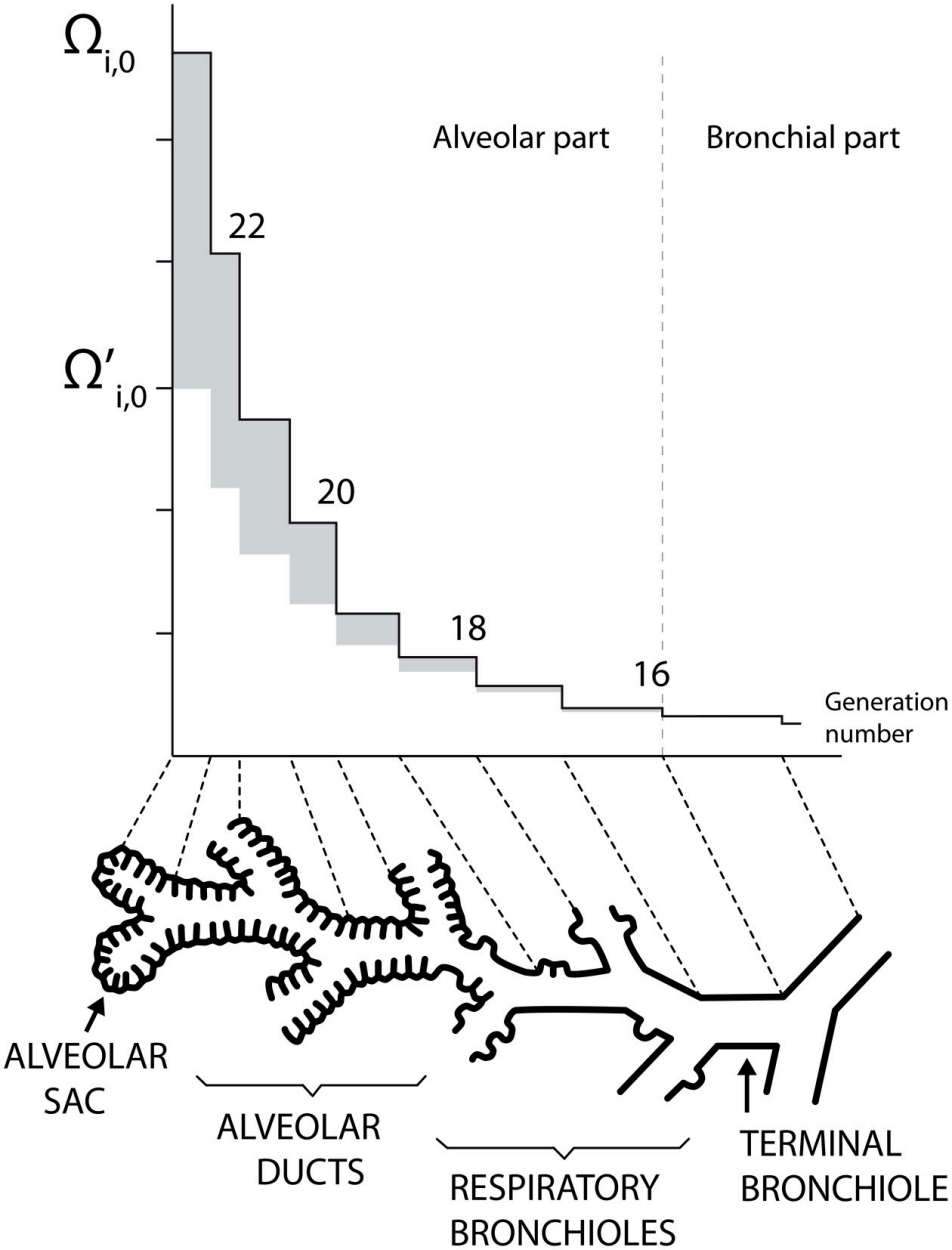
**Schematic representation of the lungs structure**. Plots of Ω_*i*, 0_ and $\Omega_{i,0}^{\prime }$ as functions of the generation number (mouth is at the right). Adapted from Paiva and Engel (1987).

**Figure 2:**
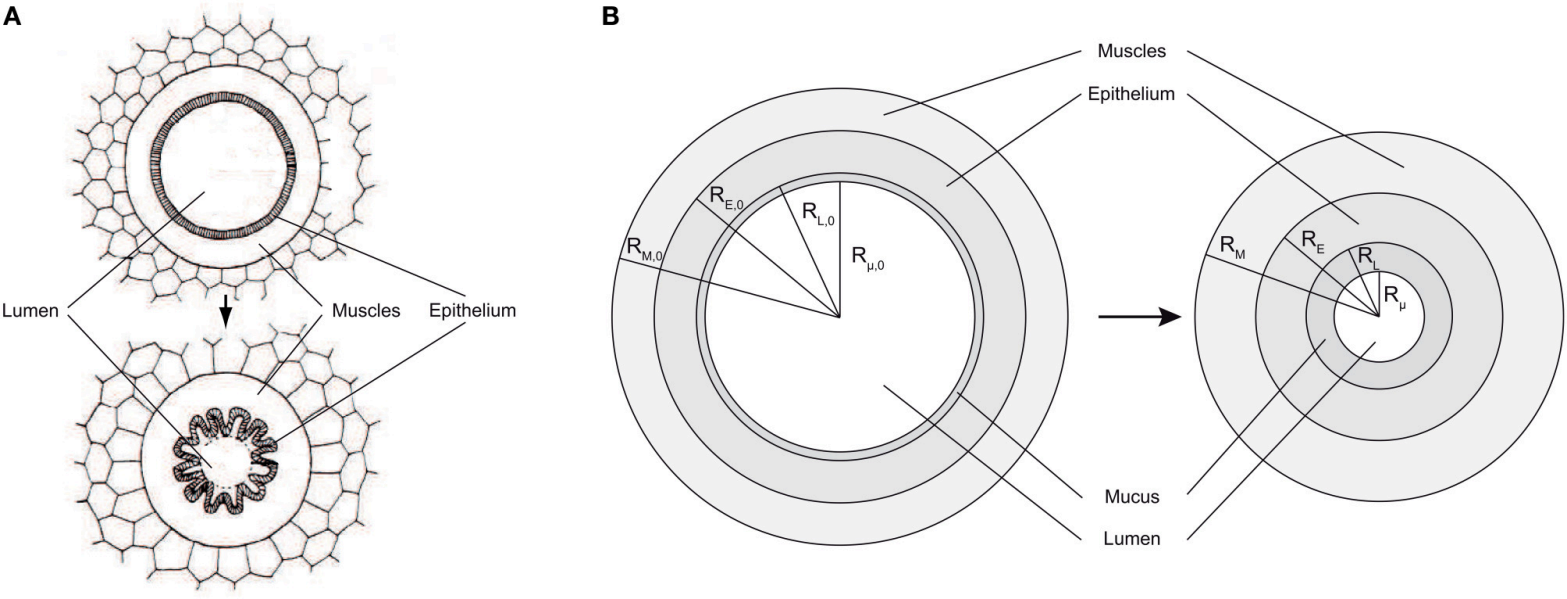
**(A)** Healthy airway (top) and constricted airway (bottom). When contracting, the smooth muscles induce a reduction of the airway lumen diameter, by wrinkling of the epithelial layer. Adapted from Yager et al. (1989). **(B)** Schema of a an airway lateral wall, including a mucus layer. Left: healthy lungs at rest. Right: lungs at rest and after bronchoconstriction.

Human lungs are a complex organ, organized around a dichotomous tubular “tree” structure (see Figure 1), purposed to optimize the transfer of the respiratory gases (mainly O_2_ and CO_2_) from the inspired air through the mouth to the blood, and inversely. Each level in the tree structure is called a generation (see Figure 1) and the lungs are composed of 24 of these generations. The generations are numbered, starting from the trachea (number 0). The lungs can be roughly divided in two compartments (see Figure 1):

- an upper or bronchial part, composed of so-called airways, where convection is the dominant mechanism of gaseous mass transport. An airway can be seen as a hollow cylinder in which gas flows. The inner space of an airway is called the lumen. The lateral wall of an airway is composed of two tissue layers (see Figure 2B). The inner one, the epithelial layer (also simply called the epithelium), is one cell thick. It may be seen as a physical barrier between the outer world (i.e., the lumen) and the inner body. The second tissue layer, surrounding the epithelium, is the smooth muscles layer. These muscles control the airway caliber change during a respiratory cycle. These two layers are surrounded by a network of tiny blood vessels. In unhealthy lungs, an epithelial layer can also be coated by a mucus layer (see Figure 2A) (Wadsworth et al., 2012). NO is produced in the epithelium and diffuses through the layers to the blood on one side and to the airway lumen on the other side, while being partially consumed by several chemical reactions in the epithelial and muscles layers. This bronchial part is approximately composed of generations 0–16 (Weibel et al., 2005).
- a lower or alveolar part. This part of the lungs is also composed of so-called airways, but with their lateral wall garnished by expansible bags called alveoli. These alveoli are optimized for mass exchange between the air and the blood. In this part of the lung, the inner space of an airway is also called the lumen (by definition, it does not include the inner space of the alveoli). Diffusion is the dominant mechanism of gaseous mass transport in this part of the lungs. NO is produced in the epithelial cells present in the wall of the alveoli. This alveolar part begins approximately at generation 17 (Weibel et al., 2005).

Multiple models have been developed in order to characterize the NO transport in the lungs, owing to their complex structure. Indeed, direct measurements in the lungs are difficult and therefore these models are valuable tools to interpret experimental data such as the FE_NO_.

In the first developed models, the lungs are divided in two perfectly mixed compartments, a rigid bronchial compartment and an expansible alveolar compartment. These models are able to reproduce some observed features of the pulmonary NO transport (Hyde et al., 1997; Tsoukias and George, 1998; Pietropaoli et al., 1999; Jörres, 2000).

As a step beyond these two-compartments models, a variety of so-called morphological models have been developed. In a morphological model, at the opposite of a two-compartments model, each generation of the lungs is represented and characterized by its number of airways, by the total volume of the lumen of its airways and, in the alveolar zone, by the total inner volume of its alveoli. It is assumed that each division in the tree structure of the lungs generates two identical airways (Paiva and Engel, 1987; Van Muylem et al., 2003). Furthermore, the NO axial transport in a generation is described by considering both convection and axial diffusion; such a description is not included in the two-compartments models. In the morphological models developed to date, the NO exchange rate between the gas and the surrounding tissues is simply expressed, in each generation, by partitioning experimentally determined total exchange rates (one for the bronchial part, one for the alveolar part).

In this work, a new morphological model of the NO transport in the human lungs is developed and used. When compared to models published previously, several new features are introduced.

First, for the generations 0 to 18, the model includes a detailed description of the lateral wall of the airways. Diffusion-reaction equations for the NO are written in the layers composing an airway wall (including a possible mucus layer). For a given airway, solving these equations allows expressing the NO exchange rate between the epithelium and the lumen of this airway as a function of fundamental parameters, such as the NO volumetric production rate in the epithelium, the airway and layers geometry. Thus, in our model and for generations 0–18, this exchange rate is not obtained by partitioning an experimentally determined total exchange rate. In the generations 19–23, the lateral wall of an airway is almost totally composed of alveoli (see Figure 1). Hence, this layered airway lateral wall representation is not relevant for these generations (Weibel et al., 2005).

Second, in our model, we also include the possibility to modulate the cross section of the airways in one or several generations of the upper part of the lungs and, therefore, to simulate bronchoconstriction (BC, the constriction of the airways in the lungs due to the tightening of muscles) which is a feature of multiple pathologies, as for instance asthma. In our model, when BC is imposed in a generation, the thicknesses of the epithelial and muscles layers of the corresponding airways are reevaluated, based on volume conservation. The thickness of a possible mucus layer is also reevaluated. Thus, by combining the possible modulation of the cross section of the airways in one or several generations and the layered description of the lateral wall of these airways, our model is able to take into account the influence of a BC on the NO exchange rate between the epithelium and the lumen of an airway in these generations, at the opposite of previous models.

Third, our model is written in a dimensionless form. This simplifies, accelerates and stabilizes the numerical calculations, when compared to previous models. It also brings out typical dimensionless numbers used in fluid mechanics, such as the Péclet number, and in chemical engineering, such as the Hatta number. Evaluation of these dimensionless numbers allows understanding the relative influence of different phenomena on the NO concentration profile in the lungs.

These new features allow our model to produce new insights into the NO transport in the human lungs, especially regarding the influence of BC on the FE_NO_.

In this paper, the developed model is first presented. Then, the model is used to discuss some features of the NO transport in healthy and unhealthy lungs. In particular, simulation results are compared with experimental information available in the literature. Moreover, using the model, the expected influence of the localization of BC and of its extent on the FE_NO_ is also discussed. Finally, two major assumptions at the basis of the model are discussed.

## 2. Model development

### 2.1. Geometrical considerations

#### 2.1.1. Definitions

The notations introduced in this subsection are applicable to any lungs, whether they are healthy or not.

As for the morphological models developed previously, it is assumed that each division in the tree structure of the lungs generates two identical airways, even when mucus is present and after a possible BC (Paiva and Engel, 1987; Van Muylem et al., 2003).

*L*_*i*_ is defined as the axial length of the airways in generation i. Ω_*i*_(*t*) is defined such that Ω_*i*_(*t*)*L*_*i*_ is the total gas volume in generation i (volume of the lumen of the airways and inner volume of the alveoli), at time *t*. Hence, Ω_*i*_(*t*) can be seen as the total cross-sectional area of generation i, at time *t*. $\Omega_{i}^{\prime }\left(t\right)$ is defined such that $\Omega_{i}^{\prime }\left(t\right)L_{i}$ is the total gas volume in the lumen of the airways in generation i, at time *t*. Hence, $\Omega_{i}^{\prime }\left(t\right)$ can be seen as the total flow cross-sectional area in generation i, at time *t*. Indeed, as the alveoli are being closely separated from each other, the axial transport in an airway occurs only through its lumen (Paiva, 1972, 1973). As a consequence of these definitions, Ω_alv, *i*_(*t*), defined by $\Omega_{\text{alv},i}\left(t\right)=\Omega_{i}\left(t\right)-\Omega_{i}^{\prime }\left(t\right)$, is such that Ω_alv, *i*_(*t*)*L*_*i*_ is the total inner volume of the alveoli in generation i, at time *t*.

As mentioned previously, for the generations 0–18, the model includes a detailed description of the lateral wall of the airways. Such a wall is composed of two tissue layers: the epithelial layer and the muscles layer. In unhealthy lungs, an epithelial layer can also be coated by a mucus layer

The airways in generations 0–18 are considered to be perfectly axisymmetric (Weibel et al., 2005). *R*_*L, i*_(*t*) is defined as the distance between the center of an airway in generation i and the inner surface of the epithelial layer of its wall, at time *t* (see Figure 2B). *R*_*E, i*_(*t*) is defined as the distance between the center of an airway in generation i and the outer surface of the epithelial layer of its wall, at time *t* (see Figure 2B). *R*_*M, i*_(*t*) is defined as the distance between the center of an airway in generation i and the outer surface of the muscles layer of its wall, at time *t* (see Figure 2B). *R*_μ, *i*_(*t*) is defined as the distance between the center of an airway in generation i and the inner surface of a possible mucus layer coating its wall, at time *t* (see Figure 2B). Finally, δ_*M, i*_(*t*), δ_*E, i*_(*t*) and δ_μ, *i*_(*t*) are defined as the thicknesses of the muscles layer, the epithelial layer and the possible mucus layer of the wall of the airways in generation i, at time *t*, respectively. They can be calculated as follows: δ_*M, i*_(*t*) = *R*_*M, i*_(*t*)−*R*_*E, i*_(*t*), δ_*E, i*_(*t*) = *R*_*E, i*_(*t*)−*R*_*L, i*_(*t*) and δ_μ, *i*_(*t*) = *R*_*L, i*_(*t*)−*R*_μ, *i*_(*t*).

As mentioned previously, in the generations 19–23, the lateral wall of an airway is almost totally composed of alveoli. Hence, the three-layers airway lateral wall representation is not relevant for these generations. Moreover, the alveoli are not surrounded by muscles and their inner surface cannot be coated with mucus, even in unhealthy lungs. Therefore, even if epithelial cells are found in the walls of each alveolus, the use of the terms “epithelial layer,” “muscles layer,” and “mucus layer” (and the use of the associated notations) is only restricted to the airways in generations 0–18.

For generations 0–18, if no mucus is present in generation i, *S*_air, *i*_(*t*) is defined as the total exchange surface between the epithelium and the lumen in this generation, at time *t*. If mucus is present in generation i, *S*_air, *i*_(*t*) is defined as the total exchange surface between the mucus and the lumen in this generation, at time *t*. For generations 19–23, *S*_air, *i*_(*t*) is set to zero, as the lateral wall of the airways in these generations is almost totally composed of alveoli. The total inner surface of the alveoli in generation i at time *t* is written *S*_alv, *i*_(*t*).

#### 2.1.2. Lungs at rest

In our model, any lungs at rest are seen as healthy lungs at rest in which some alterations may have occurred.

For healthy lungs at rest, Ω_*i*_(*t*), $\Omega_{i}^{\prime }\left(t\right)$ and Ω_alv, *i*_(*t*) are written Ω_*i*, 0_, $\Omega_{i,0}^{\prime }$ and Ω_alv, *i*, 0_, respectively. The values of *L*_*i*_, Ω_*i*, 0_ and $\Omega_{i,0}^{\prime }$ used in this paper are derived from the morphometric model of Weibel (Weibel, 1963). They are provided in Table 1, along with the numerical values of some other parameters of the model developed in this work.

**Table 1:** **Numerical values of some parameters of the model developed in this paper, derived from the morphometric model of Weibel (Weibel, 1963)**.

**i**	***L*_*i*_ (cm)**	**Lumen diameter = $\sqrt{\frac{{4\Omega }_{i,0}^{\prime }}{\pi 2^{i}}}$ (cm)**	**Ω_*i*, 0_ (cm^2^)**	**$\Omega_{i,0}^{\prime }$ (cm^2^)**	**α_max_**	**Pe**
0	12.00	1.53	1.83	1.83	0.892	15083
1	4.76	1.03	1.68	1.68	0.869	6522
2	1.90	0.67	1.54	1.54	0.841	2848
3	0.76	0.48	1.44	1.44	0.809	1213
4	1.26	0.38	1.79	1.79	0.785	1622
5	1.06	0.30	2.24	2.24	0.760	1088
6	0.90	0.24	2.86	2.86	0.733	726
7	0.76	0.19	3.68	3.68	0.704	476
8	0.64	0.158	5.02	5.02	0.676	294
9	0.54	0.131	6.90	6.90	0.647	180
10	0.46	0.110	9.67	9.67	0.618	110
11	0.38	0.094	14.15	14.15	0.591	61.9
12	0.32	0.080	20.79	20.79	0.563	35.5
13	0.26	0.072	33.56	33.56	0.542	17.85
14	0.20	0.062	50.13	50.13	0.514	9.19
15	0.20	0.056	81.56	81.56	0.493	5.65
16	0.16	0.050	130	130	0.470	2.84
17	0.14	0.046	249	217	0.451	1.49
18	0.12	0.043	530	385	0.439	0.72
19	0.1	0.041	1140	683		0.34
20	0.08	0.037	3069	1155		0.16
21	0.06	0.038	6810	2324		0.059
22	0.06	0.036	15015	4244		0.033
23	0.06	0.036	30155	8516		0.016

Ω_*i*, 0_ and $\Omega_{i,0}^{\prime }$ are presented as functions of the generation number in Figure 1. This figure illustrates the increase of Ω_*i*, 0_ and $\Omega_{i,0}^{\prime }$ along generations, which graphically form *trumpet-shape*-like plots. On this figure, the gray zones correspond to the values of Ω_alv, *i*, 0_.

The total gas volume in healthy lungs at rest (*V*_0_) can be calculated as $V_{0}=\overset{n}{\underset{i=0}{\sum }}\Omega_{i,0}L_{i}$. Using the data given in Table 1, a volume of 3700 ml is calculated.

For lungs at rest and not impacted by BC, *R*_*L, i*_(*t*), *R*_*E, i*_(*t*) and *R*_*M, i*_(*t*) are written *R*_*L, i*, 0_, *R*_*E, i*, 0_ and *R*_*M, i*, 0_, respectively. According to the fact that the airways in generations 0–18 are considered to be perfectly axisymmetric, *R*_*L, i*, 0_ can be calculated as follows: $R_{L,i,0}=\sqrt{\frac{\Omega_{i,0}^{\prime }}{\pi 2^{i}}}$. For lungs at rest and not impacted by BC, the thicknesses of the epithelial layers and of the muscles layers are written δ_*E*, 0_ and δ_*M*, 0_, respectively. These thicknesses are assumed independent of the considered generation. *R*_*E, i*, 0_ and *R*_*M, i*, 0_ can be thus calculated as follows: *R*_*E, i*, 0_ = *R*_*L, i*, 0_ + δ_*E*, 0_ and *R*_*M, i*, 0_ = *R*_*E, i*, 0_ + δ_*M*, 0_.

As mentioned previously, in unhealthy lungs, for instance in asthmatic patients, an epithelial layer can be coated with a significant mucus layer (Farmer and Hay, 1991). Actually, even in healthy lungs, there is a very thin mucus layer coating the epithelial layer of the airways in the first generations, but it is so thin that it does not influence the NO transport in the lungs (Bhaskar et al., 1985). This mucus layer is produced by dedicated epithelial cells (see Figure 2A) and protects the epithelial surface from bacteria and dust. For lungs at rest and before their potential alteration by BC, the thickness of possible pathological mucus layers in the airways in the generation i is written δ_μ, *i*, 0_. *R*_μ, *i*, 0_ is defined as the distance between the center of an airway in generation i and the inner surface of a possible mucus layer coating its wall, in lungs at rest and before their potential alteration by a BC (see Figure 2A). It can be calculated as follows: *R*_μ, *i*, 0_ = *R*_*L, i*, 0_ − δ_μ, *i*, 0_. As mentioned previously, no mucus can be present in generations 19–23. Hence, δ_μ, *i*, 0_ is set to zero for i larger or equal to 19.

BC can only occur in generations 0–18. Indeed, the lateral wall of an airway in generations 19–23 is totally composed of alveoli and the alveoli are not surrounded by muscles. If BC occurs in an airway, the muscles and epithelial layers volumes in this airway are conserved. As a consequence, the epithelial layer wrinkles (see Figure 2A). It is often assumed that this wrinkling leads to a reduction of the surface available for gas exchange between the epithelial layer and the lumen. Indeed, a very small exchange is likely to occur through the parts of the epithelial layer surface that are almost in contact with each other, due to the wrinkling (Yager et al., 1989). Therefore, it can be assumed that an airway remains axisymmetric even after a BC and that this BC can be modeled by a reduction of the airway lumen diameter, with the muscles, epithelial and mucus layers thicknesses being simply increased, while their volumes are conserved (see Figure 2B). During BC in an airway, the volume of a possible mucus layer in this airway is also obviously conserved.

For lungs at rest and after their possible alteration by BC, Ω_*i*_(*t*), $\Omega_{i}^{\prime }\left(t\right)$, *R*_*L, i*_(*t*), *R*_*E, i*_(*t*), *R*_*M, i*_(*t*) and *R*_μ, *i*_(*t*) are written Ω_*i*_, $\Omega_{i}^{\prime }$, *R*_*L, i*_, *R*_*E, i*_, *R*_*M, i*_ and *R*_μ, *i*_, respectively.

The conservation of the muscles, epithelial and mucus layers volumes implies that the following equations must hold for any lungs and for 0 ≤ *i* ≤ 18:

(1) $$\begin{array}{l} \{\begin{array}{ll} \pi (R_{M,i}^{2}-R_{E,i}^{2}) & =\;\pi (R_{M,i,0}^{2}-R_{E,i,0}^{2})\\ \pi (R_{E,i}^{2}-R_{L,i}^{2}) & =\;\pi (R_{E,i,0}^{2}-R_{L,i,0}^{2})\\ \pi (R_{L,i}^{2}-R_{\mu ,i}^{2}) & =\;\pi (R_{L,i,0}^{2}-R_{\mu ,i,0}^{2}) \end{array} \end{array}$$

α_*i*_ is a measure of the extent of a possible constriction in generation i and is defined as follows:

(2) $$\begin{array}{l} R_{M,i}=\left(1-\alpha_{i}\right)R_{M,i,0} \end{array}$$

α_*i*_ is necessary equal to zero for *i*≥19.

Combining Equation (2) and Equation (1) gives:

(3) $$\begin{array}{l} \{\begin{array}{ll} R_{E,i} & =\sqrt{\left[{\left(1-\alpha_{i}\right)}^{2}-1\right]R_{M,i,0}^{2}+R_{E,i,0}^{2}}\\ R_{L,i} & =\sqrt{\left[{\left(1-\alpha_{i}\right)}^{2}-1\right]R_{M,i,0}^{2}+R_{L,i,0}^{2}}\\ R_{\mu ,i} & =\sqrt{\left[{\left(1-\alpha_{i}\right)}^{2}-1\right]R_{M,i,0}^{2}+R_{\mu ,i,0}^{2}} \end{array} \end{array}$$

Equation (3) are only valid if radicands are positive, which gives the maximal possible value of α_*i*_, written α_*i*, max_:

(4) $$\begin{array}{l} \alpha_{i,\text{max}}=1-\sqrt{1-{\left(\frac{R_{\mu ,i,0}}{R_{M,i,0}}\right)}^{2}} \end{array}$$

Values of α_*i*, max_ are given in Table 1. They are obtained considering no mucus layer (i.e., *R*_μ, *i*, 0_ = *R*_*L, i*, 0_), considering δ_*E*, 0_ = 15 μm, δ_*M*, 0_ = 30 μm, and using the values of $\Omega_{i,0}^{\prime }$ given in Table 1.

β_*i*_ is defined as 1−$\frac{R_{\mu ,i}^{2}}{R_{\mu ,i,0}^{2}}$. It is thus a measure of the possible reduction of the lumen radius of the airways in generation i (see Figure 2B). β_*i*_ can be linked to α_*i*_ as follows:

(5) $$\begin{array}{l} \alpha_{i}=1-\sqrt{1-{\left(\frac{R_{\mu ,i,0}}{R_{M,i,0}}\right)}^{2}\beta_{i}} \end{array}$$

β_*i*_ = 1 logically implies α_*i*_ = α_*i*, max_.

As mentioned previously, the alveoli are not surrounded by muscles and they cannot by coated by mucus. Hence, the inner volume of the alveoli in a generation of unhealthy lungs is the same as the inner volume of the alveoli in this generation in the corresponding healthy lungs. Therefore, $\Omega_{i}=\Omega_{i}^{\prime }+\Omega_{\text{alv},i,0}$. For generations 0–18, $\Omega_{i}^{\prime }$ can be calculated as $\Omega_{i}^{\prime }=2^{i}\pi R_{\mu ,i}^{2}$. As mentioned previously, in generations 19–23, the airways are not affected by a possible constriction and they are not coated by mucus. Therefore, for these generations, $\Omega_{i}^{\prime }=\Omega_{i,0}^{\prime }$.

The total gas volume in lungs at rest and after their possible alteration by BC (*V*) can be calculated as $V=\overset{n}{\underset{i=0}{\sum }}\Omega_{i}L_{i}$.

#### 2.1.3. Lungs during respiratory cycles

A respiratory cycle can be divided in 3 phases: inspiration (duration: *t*^in^), a possible breath-hold (duration: *t*^bh^) and finally expiration (duration: *t*^ex^). If $Q_{0}^{\text{in}}$ is the inspiration flow rate (defined as positive) and $Q_{0}^{\text{ex}}$ is the expiration flow rate (defined as negative), $V\left(t\right)=V+Q_{0}^{\text{in}}t$ during inspiration (with *V*(*t*) the total gas volume in the lungs at time *t* and *t* = 0 at the beginning of the inspiration phase), $V\left(t\right)=V+Q_{0}^{\text{in}}t^{\text{in}}$ during breath-hold and $V\left(t\right)=V+Q_{0}^{\text{in}}t^{\text{in}}+Q_{0}^{\text{ex}}t$ during expiration (with *t* = 0 at the beginning of the expiration phase).

In normal breathing conditions, pre-inspiratory and post-expiratory lungs volumes are equal. Therefore, the following equality must hold: $Q_{0}^{\text{in}}t^{\text{in}}=-Q_{0}^{\text{ex}}t^{\text{ex}}$.

During inspiration and expiration, it is usually assumed that the length of each airway does not change. Therefore, the following equations can be written at any time:

(6) $$\begin{array}{l} \frac{V\left(t\right)}{V}=\frac{\Omega_{i}\left(t\right)}{\Omega_{i}}=\frac{\Omega_{i}^{\prime }\left(t\right)}{\Omega_{i}^{\prime }}=\frac{\Omega_{\text{alv},i}\left(t\right)}{\Omega_{\text{alv},i,0}} \end{array}$$

During an inspiration phase, as $V\left(t\right)=V+Q_{0}^{\text{in}}t$, Equation (6) can be rewritten as follows:

(7) $$\begin{array}{l} \frac{\Omega_{i}\left(\theta \right)}{\Omega_{i}}=\frac{\Omega_{i}^{\prime }\left(\theta \right)}{\Omega_{i}^{\prime }}=\frac{\Omega_{\text{alv},i}\left(\theta \right)}{\Omega_{\text{alv},i,0}}=1+\text{Dil}\theta \end{array}$$

where θ = *t*/*t*^in^ is a dimensionless time (θ = 0 at the beginning of the inspiration phase and θ = 1 at the end of the phase) and Dil is a dimensionless number defined as:

(8) $$\begin{array}{l} \text{Dil}=\frac{Q_{0}^{\text{in}}}{V}t^{\text{in}} \end{array}$$

Dil is the ratio between the total inspired air volume and the initial gas volume in the lungs.

For 0 ≤ *i* ≤ 18, $\Omega_{i}^{\prime }\left(\theta \right)=2^{i}\pi {\left(R_{\mu ,i}\left(\theta \right)\right)}^{2}$. Therefore, *R*_μ, *i*_(θ) can be calculated as follows during an inspiration phase:

(9) $$\begin{array}{l} R_{\mu ,i}\left(\theta \right)=\sqrt{\frac{\left(1+\text{Dil}\theta \right)\Omega_{i}^{\prime }}{2^{i}\pi }} \end{array}$$

The alveolar part of the lungs begins approximately at generation 17. However, only a few alveoli are found in generations 17 and 18. Hence, during an inspiration phase and for 0 ≤ *i* ≤ 18, *S*_air, *i*_(θ) can be calculated as:

(10) $$\begin{array}{l} {\text{S}}_{\text{air,}i}\left(\theta \right)=2^{i}2\pi {\;R}_{\mu ,i}\left(\theta \right)L_{i}=2\sqrt{2^{i}\pi \left(1+\text{Dil}\theta \right)\Omega_{i}^{\prime }}L_{i} \end{array}$$

If the alveoli are considered as hemispheres, *S*_alv, *i*_(θ) can be calculated as follows during an inspiration phase:

(11) $$\begin{array}{l} {\text{S}}_{\text{alv},i}\left(\theta \right)=\Omega_{\text{alv},i}\left(\theta \right)L_{i}\frac{6}{{\text{d}}_{\text{alv}}}=\Omega_{\text{alv},i,0}\left(1+\text{Dil}\theta \right)L_{i}\frac{6}{{\text{d}}_{\text{alv}}} \end{array}$$

with *d*_alv_ the inner diameter of an alveolus, that is approximately equal to 200 μm (Ochs et al., 2004).

During a breath-hold phase, as $V\left(t\right)=V+Q_{0}^{\text{in}}t^{\text{in}}$, Equation (6) can be rewritten as follows:

(12) $$\begin{array}{l} \frac{\Omega_{i}\left(\theta \right)}{\Omega_{i}}=\frac{\Omega_{i}^{\prime }\left(\theta \right)}{\Omega_{i}^{\prime }}=\frac{\Omega_{\text{alv},i}\left(\theta \right)}{\Omega_{\text{alv},i,0}}=1+\text{Dil} \end{array}$$

where θ = *t*/*t*^in^ is a dimensionless time (θ = 0 at the beginning of the breath-hold phase and θ = *t*^bh^/*t*^in^ at the end of the phase).

Consequently, the following equations can be written during a breath-hold phase:

(13) $$\begin{array}{l} R_{\mu ,i}\left(\theta \right)=\sqrt{\frac{\left(1+\text{Dil}\right)\Omega_{i}^{\prime }}{2^{i}\pi }} \end{array}$$

(14) $$\begin{array}{l} {\text{S}}_{\text{air},i}(\theta )=2\sqrt{2^{i}\pi (1+\text{Dil}){\Omega^{\prime }}_{i}}L_{i},\text{for }0\leq i\leq 18 \end{array}$$

(15) $$\begin{array}{l} {\text{S}}_{\text{alv},i}\left(\theta \right)=\Omega_{\text{alv},i,0}\left(1+\text{Dil}\right)L_{i}\frac{6}{{\text{d}}_{\text{alv}}} \end{array}$$

During an expiration phase, as $V\left(t\right)=V+Q_{0}^{\text{in}}t^{\text{in}}+Q_{0}^{\text{ex}}t$ and as $Q_{0}^{\text{in}}t^{\text{in}}=-Q_{0}^{\text{ex}}t^{\text{ex}}$, Equation (6) can be rewritten as follows:

(16) $$\begin{array}{l} \frac{\Omega_{i}\left(\theta \right)}{\Omega_{i}}=\frac{\Omega_{i}^{\prime }\left(\theta \right)}{\Omega_{i}^{\prime }}=\frac{\Omega_{\text{alv},i}\left(\theta \right)}{\Omega_{\text{alv},i,0}}=1+\text{Dil}\left(1-\theta \right) \end{array}$$

where θ = *t*/*t*^ex^ is a dimensionless time (θ = 0 at the beginning of the expiration phase and θ = 1 at the end of the phase).

Consequently, the following equations can be written during an expiration phase:

(17) $$\begin{array}{l} R_{\mu ,i}\left(\theta \right)=\sqrt{\frac{\left(1+\text{Dil}\left(1-\theta \right)\right)\Omega_{i}^{\prime }}{2^{i}\pi }} \end{array}$$

(18) $$\begin{array}{l} {\text{S}}_{\text{air},i}\left(\theta \right)=2\sqrt{2^{i}\pi \left(1+\text{Dil}\left(1-\theta \right)\right)\Omega_{i}^{\prime }}L_{i},\text{for }0\leq i\leq 18 \end{array}$$

(19) $$\begin{array}{l} {\text{S}}_{\text{alv},i}\left(\theta \right)=\Omega_{\text{alv},i,0}\left(1+\text{Dil}\left(1-\theta \right)\right)L_{i}\frac{6}{{\text{d}}_{\text{alv}}} \end{array}$$

At any time, *R*_*L, i*_(θ), *R*_*E, i*_(θ) and *R*_*M, i*_(θ) can be related to *R*_μ, *i*_(θ) using volume conservation:

(20) $$\begin{array}{l} \{\begin{array}{ll} R_{L,i}(\theta ) & =\;\sqrt{R_{L,i}^{2}-R_{\mu ,i}^{2}+R_{\mu ,i}{\left(\theta \right)}^{2}}\\ R_{E,i}(\theta ) & =\;\sqrt{R_{E,i}^{2}-R_{L,i}^{2}+R_{L,i}{\left(\theta \right)}^{2}}\\ R_{M,i}(\theta ) & =\;\sqrt{R_{M,i}^{2}-R_{E,i}^{2}+R_{E,i}{\left(\theta \right)}^{2}} \end{array} \end{array}$$

### 2.2. Air flow in the lungs

In each airway, an axial coordinate *z* is introduced. Regarding an airway in the generation i, *z* = 0 at the beginning of the airway, while *z* = *L*_*i*_ at its end. In each airway, a dimensionless axial coordinate ζ is also introduced. ζ = 0 at the beginning of the airway and ζ = 1 at its end. Therefore, regarding an airway in generation i, ζ = *z*/*L*_*i*_

*Q*_*i*_(*t, z*) is defined as the air volumetric flow rate at time *t* and at position *z*, in generation i. *Q*_*i*_(*t, z*) is positive when air flows from the trachea to the alveolar part of the lungs (during inspiration) and negative when air flows from the alveolar part of the lungs to the trachea (during expiration).

The air flow in lungs can be considered as incompressible (Paiva and Engel, 1987). Therefore, considering the assumption that the length of each airway does not change during a respiratory cycle, a mass balance for the gas over a slice of the airways in generation i leads to the following equation:

(21) $$\begin{array}{l} -\frac{\partial Q_{i}}{\partial z}=\frac{d\Omega_{i}}{dt} \end{array}$$

Using Equation (6), this mass balance equation can be rewritten in a dimensionless form, for each respiratory phase:

(22) $$\begin{array}{l} \{\begin{array}{ll} \text{Inspiration: } & -\frac{\partial Q_{i}}{\partial \zeta }=Q_{0}^{\text{in}}{\text{f}}_{i}\\ \text{Breath-hold: } & -\frac{\partial Q_{i}}{\partial \zeta }=0\\ \text{Expiration: } & -\frac{\partial Q_{i}}{\partial \zeta }=Q_{0}^{\text{ex}}{\text{f}}_{i} \end{array} \end{array}$$

where ${\text{f}}_{i}=\frac{L_{i}\Omega_{i}}{V}$ is the ratio of the volume of gas in generation i to the total volume of gas in the lungs, when they are at rest.

The integration of Equation (22) gives:

(23) $$\begin{array}{l} \{\begin{array}{ll} \text{Inspiration: } & Q_{i}(\zeta )=Q_{0}^{\text{in}}\left(1-\sum_{j\;=\;0}^{i\;-\;1}{\text{f}}_{j}-{\text{f}}_{i}\zeta \right)\\ \text{Breath-hold: } & Q_{i}(\zeta )=0\\ \text{Expiration: } & Q_{i}(\zeta )=Q_{0}^{\text{ex}}\left(1-\sum_{j\;=\;0}^{i\;-\;1}{\text{f}}_{j}-{\text{f}}_{i}\zeta \right) \end{array} \end{array}$$

It appears that *Q*_*i*_ does not depend explicitly on the time *t*. Hence, according to our modeling assumptions, the air flow in the lungs is quasi-steady.

### 2.3. NO exchange

In the lungs, the major part of the NO production is thought to arise from various types of epithelial cells (Dillon et al., 1996; Jiang et al., 2009). In these cells, NO is synthesized by a group of enzymes, called NO synthases (NOS). On the other hand, NO is consumed in the tissues. Indeed, NO is a labile molecule that reacts with biological compounds such as O_2_, superoxides or metalloproteins (Gaston et al., 1994). It has been shown that the rate of NO consumption in tissues can be approximated as being of the first order in NO concentration (Tsoukias and George, 1998).

Note that NO has a great affinity with the blood hemoglobin (Hb), far more than O_2_ or even CO. Trapped by the Hb, NO becomes unavailable, so that its blood concentration can always be considered as null (Tsoukias and George, 1998).

#### 2.3.1. NO exchange between the wall of the alveoli and the gas in an airway

*J*_alv, *i*_(θ, ζ) is defined as the NO exchange flux density between the wall of the alveoli in generation i and the gas in this generation, at the dimensionless time θ and at the dimensionless position ζ, expressed in ml of gaseous NO exchanged per second and per cm^2^ of the inner surface of the alveoli. *J*_alv, *i*_ is defined as being positive when the net NO flow is going from the wall of the alveoli to the gas.

According to Van Muylem et al. (2003), *J*_alv, *i*_(θ, ζ) can be expressed as follows:

(24) $$\begin{array}{l} J_{\text{alv},i}\left(\theta ,\zeta \right)=\frac{P_{\text{alv}}}{S_{\text{alv,tot}}\left(\theta \right)}-\frac{U_{\text{alv}}}{S_{\text{alv,tot}}\left(\theta \right)}C_{i}\left(\theta ,\zeta \right) \end{array}$$

where *C*_*i*_(θ, ζ) is the NO concentration, at the dimensionless time θ and at the dimensionless position ζ in the lumen of an airway in generation i. It is expressed in ml of gaseous NO per cm^3^ of air. This concentration is assumed homogeneous in a cross-section of the airway (Paiva and Engel, 1987). $S_{\text{alv,tot}}\left(\theta \right)=\overset{23}{\underset{i\;=\;0}{\sum }}S_{\text{alv},i}\left(\theta \right)$, *P*_alv_ is the total alveolar NO production rate, expressed in ml of gaseous NO per second, and *U*_alv_ is a constant describing the NO consumption in the tissues, expressed in cm^3^ of air per second.

#### 2.3.2. NO exchange between the epithelial layer and the lumen of an airway

*J*_air, *i*_(θ, ζ) is defined as the NO exchange flux density between the epithelial layer and the lumen, at the dimensionless time θ and at the dimensionless position ζ, in an airway of generation i. It is expressed in ml of gaseous NO exchanged per second and per cm^2^ of the exchange surface between the lumen and the epithelial layer of an airway in the generation (if no mucus is present in the generation) or between the lumen and the mucus layer of an airway in the generation (if mucus is present in the generation). *J*_air, *i*_ is defined as being positive when the net NO flow is going from the airway tissues to the lumen.

We first examine the case of an airway with its epithelial layer coated with mucus.

**Figure 3:**
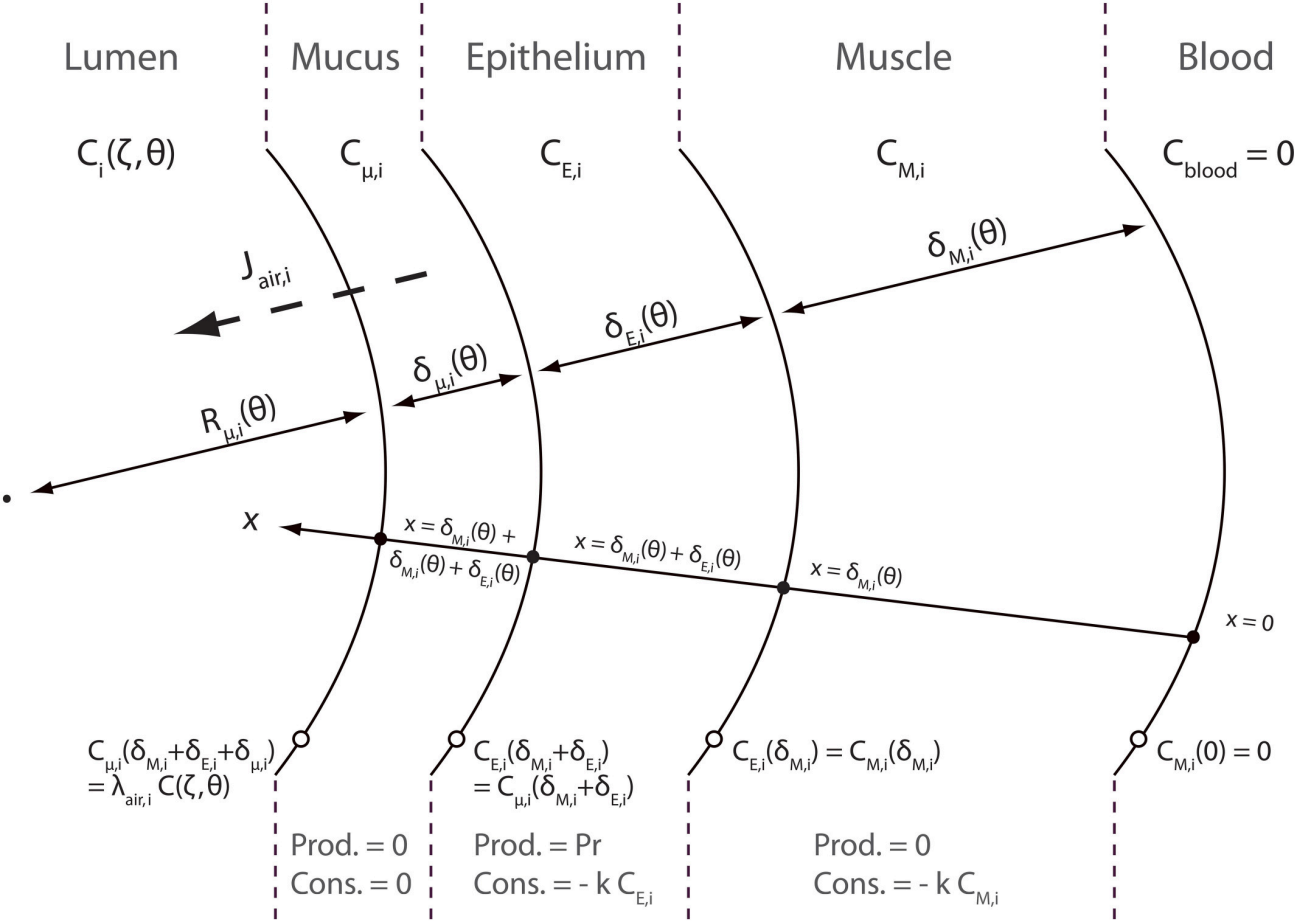
**Schematic representation of the NO transfers inside the layers of an airway wall**.

A schematic representation of the NO transfer inside the layers of an airway wall is presented in Figure 3. NO diffuses through these layers. This diffusion can be assimilated to diffusion in pure liquid water (Tsoukias and George, 1998). As mentioned previously, NO is produced and consumed in the epithelial layer. It is also consumed in the muscle layer. As NO is trapped by Hb, its concentration in the blood is null. A coordinate *x*, pointing toward the lumen, is introduced in Figure 3. *x* = 0 at the blood-muscle interface. C_μ, *i*_(*t, x*), C_*E, i*_(*t, x*) and C_*M, i*_(*t, x*) are defined as the NO concentration, at time *t* and at position *x* in the mucus layer, the epithelial layer and the muscles layer of an airway in generation i, respectively. These concentrations are expressed in moles of NO per cm^3^ of layer. They also depend on the position (*z*) in the generation, but it is not explicitly written.

In order to write NO transport equations in the layers of an airway wall, two assumptions are made. The first assumption is that the transport of NO inside these layers can be assumed quasi-steady (i.e., the time derivative terms in the transport equations can be set to zero). The second assumption is that the curvature of the layers can be neglected when establishing these transport equations. This assumption is valid if *R*_μ, *i*_(*t*) is way larger than the layers thicknesses δ_*M, i*_(*t*), δ_*E, i*_(*t*) and δ_μ, *i*_(*t*). The validity of these two assumptions is further discussed in this paper. According to these assumptions, the following transport equations can be written:

(25) $$\begin{array}{l} \{\begin{array}{l} D_{\text{NO},t}\frac{d^{2}C_{E,i}}{dx^{2}}+Pr-kC_{E,i}=0\\ D_{\text{NO},t}\frac{d^{2}C_{M,i}}{dx^{2}}-kC_{M,i}=0\\ D_{\text{NO},t}\frac{d^{2}C_{\mu ,i}}{dx^{2}}=0 \end{array} \end{array}$$

where *D*_NO, *t*_ is the diffusion coefficient of NO in pure liquid water, *k* is the tissue NO consumption rate kinetic constant and *Pr* is the NO volumetric production rate in the epithelial layer, expressed in moles of NO per second and per cm^3^ of the epithelial layer. It is assumed in this work that *Pr* takes the same value in any generation.

In the mucus layer, at the interface with the lumen, the NO concentration is assumed to be at equilibrium with the NO concentration in the lumen. Hence, the following equation can be written:

(26) $$\begin{array}{l} C_{\mu ,i}\left(t,\delta_{M,i}\left(t\right)+\delta_{E,i}\left(t\right)+\delta_{\mu ,i}\left(t\right)\right)=\lambda_{\text{t:air}}C_{i}\left(t,z\right) \end{array}$$

where λ_t:air_ is a thermodynamic equilibrium constant, calculated from the Henry's constant of the NO in water, the soft tissues here being approximated as having the same chemical properties as water. At 37°C and 1 atm, $\lambda_{\text{t:air}}=1.64\times 10^{-6}\frac{\text{molNO in tissues}}{{\text{cm}}^{3}\text{NO in airways}}$ (National Research Council (U.S.), 1928; Tsoukias and George, 1998). As mentioned previously, *C*_*i*_(*t, z*) is the NO concentration, at time *t* and at position *z* in the lumen of an airway in generation i, expressed in ml of gaseous NO per cm^3^ of air. As mentioned previously, this concentration is assumed homogeneous in a cross-section of the airway.

The other boundary conditions completing Equation (25) are:

(27) $$\begin{array}{l} \{\begin{array}{l} C_{M,i}(t,0)=0\\ C_{E,i}(t,\delta_{M,i}(t))=C_{M,i}(t,\delta_{M,i}(t))\\ C_{E,i}(t,\delta_{M,i}(t)+\delta_{E,i}(t))=C_{\mu ,i}(t,\delta_{M,i}(t)+\delta_{E,i}(t))\\ \frac{dC_{M,i}}{dx}{|}_{t,x\;=\;\delta_{M,i}(t)}=\frac{dC_{E,i}}{dx}{|}_{t,x\;=\;\delta_{M,i}(t)}\\ \frac{dC_{E,i}}{dx}{|}_{t,x\;=\;\delta_{M,i}(t)\;+\;\delta_{E,i}(t)}=\frac{dC_{\mu ,i}}{dx}{|}_{t,x\;=\;\delta_{M,i}(t)\;+\;\delta_{E,i}(t)} \end{array} \end{array}$$

According to these transport equations, *J*_air, *i*_(θ, ζ) can be calculated as:

(28) $$\begin{array}{l} J_{\text{air},i}(\theta ,\zeta )=-\gamma D_{\text{NO},t}\frac{dC_{\mu ,i}}{dx}{|}_{\theta ,x\;=\;\delta_{\mu ,i}(\theta )\;+\;\delta_{E,i}(\theta )\;+\;\delta_{M,i}(\theta )} \end{array}$$

where, according to the units of *J*_air, *i*_ and *C*_μ, *i*_ and using the ideal gas law, $\gamma =2.545\times 10^{4}\frac{{\text{cm}}^{3}}{\text{mol}}$ (at atmospheric pressure and at a temperature of 319.15 K). γ is used to correctly express *J*_air, *i*_(θ, ζ) in $\frac{\text{cm}}{\text{s}}$.

Solving the transport Equation (25) with boundary conditions 26 and 27 allows obtaining the following expression for *J*_air, *i*_(θ, ζ):

(29) $$\begin{array}{l} J_{\text{air},i}(\theta ,\zeta )=Pr\sqrt{\frac{D_{\text{NO},t}}{k}}\frac{\left(e^{{\text{Hã}}_{i}}-1\right)\left(-1+e^{2{\text{Ha}}_{i}+{\text{Hã}}_{i}}\right)}{-1+{\text{Mu}}_{i}+e^{2({\text{Ha}}_{i}+{\text{Hã}}_{i})}(1+{\text{Mu}}_{i})}\gamma \\ \;-\lambda_{\text{t:air}}\sqrt{kD_{\text{NO},t}}\frac{1+e^{2({\text{Ha}}_{i}+{\text{Hã}}_{i})}}{-1+{\text{Mu}}_{i}+e^{2({\text{Ha}}_{i}+{\text{Hã}}_{i})}(1+{\text{Mu}}_{i})}\gamma C_{i}(\theta ,\zeta ) \end{array}$$

where

(30) $$\begin{array}{l} {\text{Ha}}_{i}=\sqrt{\frac{k}{D_{\text{NO},t}}}\delta_{M,i}\left(\theta \right)\text{,}{\text{Hã}}_{i}=\sqrt{\frac{k}{D_{\text{NO},t}}}\delta_{E,i}\left(\theta \right)\text{and}\\ {\text{Mu}}_{i}=\sqrt{\frac{k}{D_{\text{NO},t}}}\delta_{\mu ,i}\left(\theta \right) \end{array}$$

Ha_*i*_ and Hã_*i*_ compare a reaction characteristic time ($\frac{1}{k}$) to diffusion characteristic times $\left(\frac{\delta_{\mu ,i}^{2}\left(\theta \right)}{D_{\text{NO},t}}\text{and}\frac{\delta_{E,i}^{2}\left(\theta \right)}{D_{\text{NO},t}}\right)$. These dimensionless numbers are usually called Hatta numbers in chemical engineering. Note that these three dimensionless numbers depend on the time, thus instantaneously linking *J*_air, *i*_ to the respiratory cycle variations. The functions *C*_*M, i*_(*t, z*), *C*_*E, i*_(*t, z*) and *C*_μ, *i*_(*t, z*), solutions of Equation (25) with boundary conditions 26 and 27 are given in **Appendix A**.

It can be observed in Equation (29) that, similarly to *J*_alv, *i*_(θ, ζ), *J*_air, *i*_(θ, ζ) is composed of two terms: a positive production term proportional to *Pr* and a negative consumption term proportional to the NO concentration in the lumen.

It is important to highlight that, at the contrary to *J*_alv, *i*_(θ, ζ), *J*_air, *i*_(θ, ζ) is modified by a possible constriction in generation i. Indeed, as mentioned previously, constriction increases the thickness of the epithelial, muscles and mucus layers. This modifies the NO diffusion lengths through the layers, and therefore *J*_air, *i*_.

In the case of an airway without mucus, a similar reasoning allows obtaining the following equation to calculate *J*_air, *i*_(θ, ζ):

(31) $$\begin{array}{l} J_{\text{air},i}(\theta ,\zeta )=Pr\sqrt{\frac{D_{\text{NO},t}}{k}}\frac{\left(e^{{\text{Hã}}_{i}}-1\right)\left(-1+e^{2{\text{Ha}}_{i}+{\text{Hã}}_{i}}\right)}{-1+e^{2({\text{Ha}}_{i}+{\text{Hã}}_{i})}}\gamma \\ \;-\lambda_{\text{t:air}}\sqrt{kD_{\text{NO},t}}\frac{1+e^{2({\text{Ha}}_{i}+{\text{Hã}}_{i})}}{-1+e^{2({\text{Ha}}_{i}+{\text{Hã}}_{i})}}\gamma C_{i}(\theta ,\zeta ) \end{array}$$

### 2.4. Transport of gaseous NO in the lumen

As mentioned previously, the alveoli are being closely separated from each other. Therefore, the axial transport in an airway occurs only through its lumen. According to this, a mass balance for the gaseous NO over a slice of the airways in generation i leads, considering convective and diffusive transport, and considering Equations (6) and (21), to the following equation:

(32) $$\begin{array}{l} \frac{\partial C_{i}}{\partial t}=-\frac{Q_{i}(z)}{\Omega_{i}(t)}\frac{\partial C_{i}}{\partial z}+D_{\text{NO,air}}\frac{{\Omega^{\prime }}_{i}}{\Omega_{i}}\frac{\partial^{2}C_{i}}{\partial z^{2}}\\ \;+\frac{1}{L_{i}\Omega_{i}(t)}\left(J_{\text{alv},i}(z,t)S_{\text{alv},i}(t)+J_{\text{air},i}(z,t)S_{\text{air},i}(t)\right) \end{array}$$

where D_NO, air_ is the diffusion coefficient of NO in air.

Using Equations (7), (8), (12), (16), and (23), this transport equation can be rewritten in its dimensionless form, for each phase of a respiratory cycle:

#### 2.4.1. Inspiration phase

(33) $$\begin{array}{l} \frac{\partial C_{i}}{\partial \theta }=\frac{\text{Dil}}{{\text{f}}_{i}}(-\frac{1-\overset{i-1}{\underset{j=1}{\sum }}{\text{f}}_{j}-{\text{f}}_{i}\zeta }{1+\text{Dil}\theta }\frac{\partial C_{i}}{\partial \zeta }+\frac{1}{{\text{Pe}}_{i}}\frac{\partial^{2}C_{i}}{\partial \zeta^{2}}\\ \;+\frac{1}{1+\text{Dil}\theta }\left[J_{\text{alv},i}\left(\theta ,\zeta \right)\frac{S_{\text{alv},i}\left(\theta \right)}{Q_{0}^{\text{in}}}+J_{\text{air},i}\left(\theta ,\zeta \right)\frac{S_{\text{air},i}\left(\theta \right)}{Q_{0}^{\text{in}}}]\right),\\ \;0\leq \theta \leq 1 \end{array}$$

#### 2.4.2. Breath-hold phase

(34) $$\begin{array}{l} \frac{\partial C_{i}}{\partial \theta }=\frac{\text{Dil}}{{\text{f}}_{i}}(\frac{1}{{\text{Pe}}_{i}}\frac{\partial^{2}C_{i}}{\partial \zeta^{2}}+\frac{1}{1+\text{Dil}}\\ \;\left[J_{\text{alv},i}\left(\theta ,\zeta \right)\frac{S_{\text{alv},i}\left(\theta \right)}{Q_{0}^{\text{in}}}+J_{\text{air},i}\left(\theta ,\zeta \right)\frac{S_{\text{air},i}\left(\theta \right)}{Q_{0}^{\text{in}}}]\right),\\ \;0\leq \theta \leq \frac{t^{\text{bh}}}{t^{\text{in}}} \end{array}$$

#### 2.4.3. Expiration phase

(35) $$\begin{array}{l} \frac{\partial C_{i}}{\partial \theta }=\frac{\text{Dil}}{{\text{f}}_{i}}(\frac{1-\sum_{j=1}^{i-1}{\text{f}}_{j}-{\text{f}}_{i}\zeta }{1+\left(1-\theta \right)\text{Dil}}\frac{\partial C_{i}}{\partial \zeta }\\ \;+\frac{1}{{\text{Pe}}_{i}}\frac{\partial^{2}C_{i}}{\partial \zeta^{2}}\frac{t^{\text{ex}}}{t^{\text{in}}}+\frac{1}{1+\left(1-\theta \right)\text{Dil}}\frac{t^{\text{ex}}}{t^{\text{in}}}\\ \;\left[J_{\text{alv},i}\left(\theta ,\zeta \right)\frac{S_{\text{alv},i}\left(\theta \right)}{Q_{0}^{\text{in}}}+J_{\text{air},i}\left(\theta ,\zeta \right)\frac{S_{\text{air},i}\left(\theta \right)}{Q_{0}^{\text{in}}}]\right),\\ \;0\leq \theta \leq 1 \end{array}$$

These equations are completed by boundary conditions expressing the continuity of the gaseous NO concentration and diffusion flux at the junctions between generations. The NO diffusion flux is assumed null at the end of the last generation. During inspiration, the gaseous NO concentration at the mouth (beginning of the first generation) is set to zero. During breath-hold and expiration, the NO diffusion flux is assumed null at the mouth (pure convective transport).

Pe_*i*_ appearing in these equations is defined as:

(36) $$\begin{array}{l} {\text{Pe}}_{i}=\frac{L_{i}Q_{0}^{\text{in}}}{D_{\text{NO,air}}\Omega_{i}^{\prime }} \end{array}$$

Pe_*i*_ is a dimensionless number, usually called the Péclet number, defined for each generation. Pe_*i*_ is the ratio of a characteristic time of the axial transport of gaseous NO by convection in generation i ($\Omega_{i}^{\prime }L_{i}/Q_{0}^{\text{in}}$) to a characteristic time of the axial transport of gaseous NO by diffusion in generation i ($L_{i}^{2}/D_{\text{NO,air}}$).

### 2.5. Model summary

The model developed in this paper is characterized by several input parameters. These include geometrical parameters of the healthy lungs at rest (*L*_*i*_, $\Omega_{i,0}^{\prime }$, Ω_*i*, 0_, δ_*E*, 0_, δ_*M*, 0_ and *d*_alv_), parameters related to possible alterations of the lungs (α_*i*_ or β_*i*_ and δ_μ, *i*, 0_) and other parameters listed in Table 2. Referenced values of most of these parameters are given in Tables 1, 2. From one person to another, the values of several of these parameters show a great variability. Hence, several data presented in Tables 1, 2 should rather be seen as orders of magnitude. This large variability makes difficult a precise comparison between any model of NO transport in the lungs and experimental data. At best, such a model could be used to analyze trends observed in large amount of patients and give insights into the mechanisms governing NO transport in unhealthy lungs and, therefore, into the link between alterations of the lungs and modifications of the FE_NO_. However, in most of the situations, it could not be suited to give a precise diagnostic to a single patient.

**Table 2:** **Referenced values of several parameters of the model**.

**Parameter**	**Value**	**Units**	**References**
*d*_alv_	0.0200	cm	Ochs et al., 2004
*D*_NO, air_	0.217	cm^2^ s^−1^	Van Muylem et al., 2003
*D*_NO, *t*_	3.3 × 10^−5^	cm^2^ s^−1^	Tsoukias and George, 1998
δ_*E*, 0_	0.0015	cm	Farmer and Hay, 1991
δ_*M*, 0_	0.0030	cm	Farmer and Hay, 1991
γ	2.545 × 10^4^	cm^3^ mol^−1^	Adapted
*k*	2.001	s^−1^	Adapted from (Tsoukias and George, 1998)
λ_t:air_	1.64 × 10^−6^	molNO cm^−3^	Adapted from (National Research Council (U.S.), 1928; Tsoukias and George, 1998)
*Pr*	5.17 × 10^−12^	molNO cm^−3^ s^−1^	Adapted from (Tsoukias and George, 1998)
*P*_alv_	3.167 × 10^−6^	mlNO s^−1^	Pietropaoli et al., 1999
*U*_alv_	1558	cm^3^ s^−1^	Pietropaoli et al., 1999
${\text{Q}}_{0}^{\text{in}}$	500	ml s^−1^	Kerckx and Van Muylem, 2009
${\text{Q}}_{0}^{\text{ex}}$	−50	ml s^−1^	American Thoracic Society and European Respiratory Society, 2005
*t*^in^	2	s	Kerckx and Van Muylem, 2009
*t*^bh^	0	s	Kerckx and Van Muylem, 2009
*t*^ex^	20	s	American Thoracic Society and European Respiratory Society, 2005

The values of $Q_{0}^{\text{in}}$ and *t*^in^ provided in Table 2 are average values for adults, when they inflate their lungs to the maximum, at a normal pace (Kerckx and Van Muylem, 2009). Corresponding values of Pe_*i*_ for healthy lungs are provided in Table 1. They were obtained using the values of *L*_*i*_, $\Omega_{i}^{\prime }$ and *D*_NO, air_ given in Tables 1, 2. The values of $Q_{0}^{\text{ex}}$, *t*^bh^ and *t*^ex^ provided in Table 2 are international standard guidelines for FE_NO_ measurement experiments (i.e., a patient is asked to try to realize these values of $Q_{0}^{\text{ex}}$, *t*^bh^ and *t*^ex^ when he performs a FE_NO_ measurement experiment) (American Thoracic Society and European Respiratory Society, 2005). Together, the values of $Q_{0}^{\text{in}}$, $Q_{0}^{\text{ex}}$, *t*^in^, *t*^bh^ and *t*^ex^ provided in Table 2 define what we call a classical respiratory cycle during a FE_NO_ measurement experiment.

Once the values of the input parameters of the model defined, the geometrical properties of the lungs at rest and during respiratory cycles can be successively determined using the equations presented previously. Then, the transport Equations (33), (34), or (35) are solved for each generation, depending on the considered respiratory phase, with *J*_air, *i*_ calculated with Equation (29) or Equation (31), depending on the presence of mucus or not in the considered generation, and *J*_alv, *i*_ calculated with Equation (24). To be solved numerically, these equations are discretized using a first order upwind scheme for their convection term and a second order centered scheme for their diffusion term. To simulate a full respiratory cycle, *C*_*i*_(θ, ζ) is set to zero in each generation at the beginning of the inspiration phase. At the beginning of the breath-hold phase, the calculated values of *C*_*i*_(θ, ζ) at the end of the inspiration phase are used as initial conditions. Similarly, at the beginning of the expiration phase, the calculated values of *C*_*i*_(θ, ζ) at the end of the breath-hold phase are used as initial conditions. It is important to point out that, due the the use of the dimensionless coordinate ζ = *z*/*L*_*i*_ in each generation, these transport equations are solved between ζ = 0 and ζ = 1 in each generation. The same number of spatial discretization points is used in each generation. Simulations are performed using Wolfram Mathematica 7. A mathematica notebook “NO transport in lungs.nb” is provided as a Supplementary Material. Two functions are defined in this notebook : “modunhealthylungs” and “modhealthylungs.” The first one can be used to simulate a full respiratory cycle in an unhealthy lungs, while the second can be used to simulate a full respiratory cycle in a healthy lungs. These functions have been used to generate all the results presented in this paper.

## 3. Model capabilities

### 3.1. Healthy lungs

In this section, the model is used to discuss some features of the NO transport in healthy lungs. In particular, it is checked that the model is able to reproduce experimental information available in the literature.

The developed model can be used to simulate a respiratory cycle, for a given set of the model parameters. Using the simulation results, the time evolution of the FE_NO_ can be reported, during the expiration phase of the cycle.

**Figure 4:**
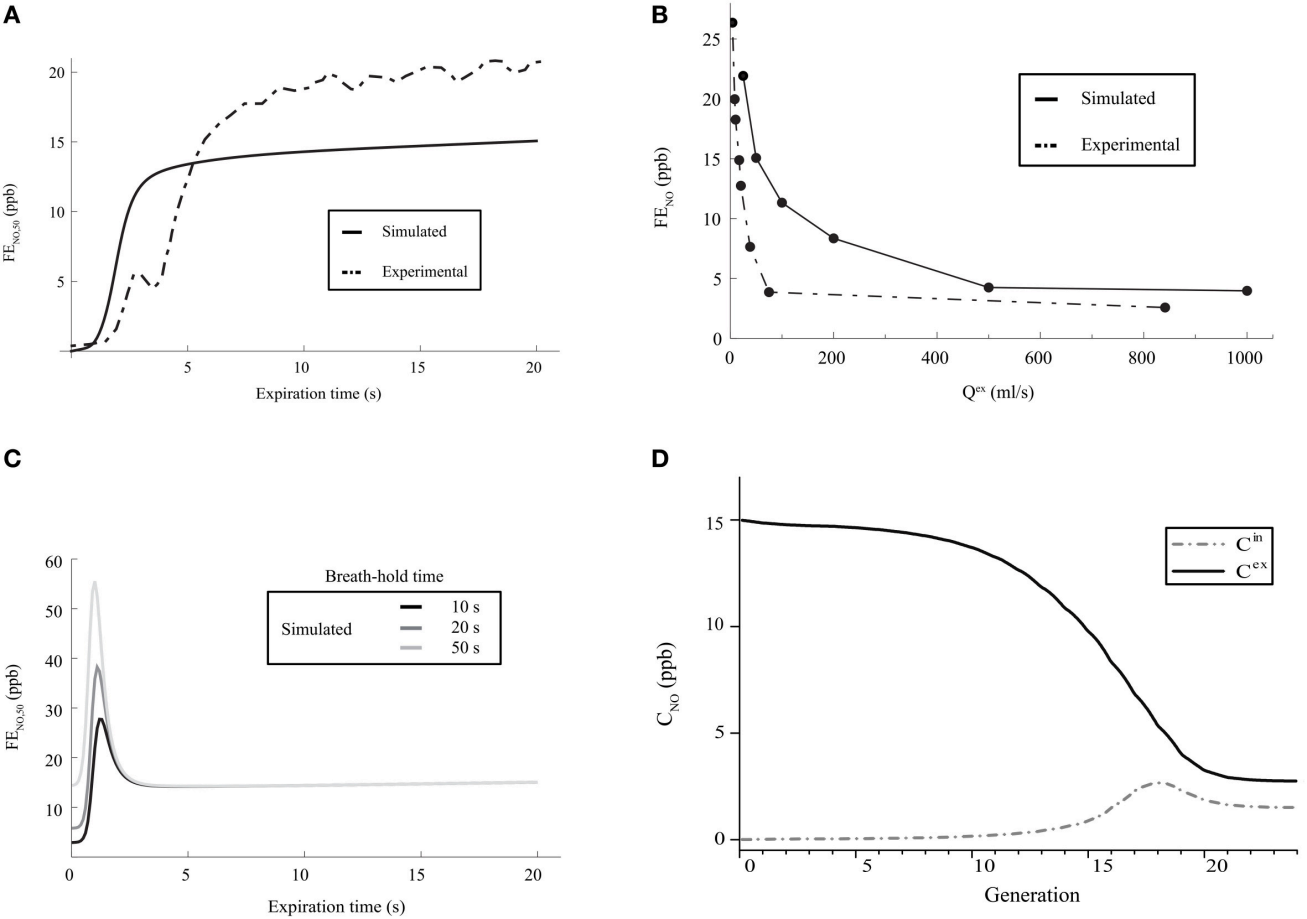
**(A)** Time evolution of the FE_NO, 50_ during the expiration phase of a classical respiratory cycle. Dashed line: measurements on a single healthy patient by Kerckx (2009). Solid line: calculated with our model (using the parameters values given in Tables 1, 2 and without considering any BC nor mucus layer). **(B)** FE_NO_ at the end of the expiration phase of a respiratory cycle, as a function of *Q*^ex^. Dashed line: measurements on a single healthy patient by Silkoff et al. (1997). Solid line: calculated with our model (using the parameters values given in Tables 1, 2 and without considering any BC nor mucus layer). **(C)** Time evolution of the FE_NO, 50_ during the expiration phase of a respiratory cycle, calculated with our model (using the parameters values given in Tables 1, 2 and without considering any BC nor mucus layer), for different values of the breath-hold duration time. **(D)** Gaseous NO concentration profile in the lungs, at the end of the inspiration phase and at the end of the expiration phase, calculated with our model (using the parameters values given in Tables 1, 2 and without considering any BC nor mucus layer). Solid line: NO concentration at the end of the expiration phase. Dashed line: NO concentration at the end of the inspiration phase.

A first characteristic of the pulmonary NO transport is related to the time evolution of the FE_NO_ during the expiration phase of a classical respiratory cycle. During such an expiration phase, the FE_NO_ is expected to rise rapidly during a few seconds, until reaching a value of 10–20 ppb. Then, it is expected to increase slowly during the rest of the expiration phase (American Thoracic Society and European Respiratory Society, 2005; Kerckx, 2009). In Figure 4A, the time evolution of the FE_NO_ during the expiration phase of a classical respiratory cycle, calculated with our model for healthy lungs (using the parameters values given in Tables 1, 2), is presented. It is observed that the expected time evolution of the FE_NO_ is indeed reproduced by the model (solid line). In Figure 4A, an example of the time evolution of the FE_NO_ measured on a single healthy patient by Kerckx (Kerckx, 2009) during the expiration phase of a classical respiratory cycle is also presented (dashed line). The value of the FE_NO_ at the end of a respiratory cycle when an expiration flow rate of 50 ml/s is realized is written FE_NO, 50_.

A second characteristic of the pulmonary NO transport is related to the link between the expiration flow rate and the value of the FE_NO_ at the end of the respiratory cycle. It has been experimentally shown that this value of the FE_NO_ decreases when the expiration flow rate increases. It is close to 15–30 ppb at a small flow rate (25 ml/s) and decreases until reaching a constant value of approximately 5 ppb at larger flow rates (Silkoff et al., 1997; Pietropaoli et al., 1999). In Figure 4B, the value of the FE_NO_ at the end of the expiration phase, calculated by the model for healthy lungs, is presented as a function of *Q*^ex^. The data presented in Tables 1, 2 have been used to generate these results. It is observed that the expected link between the value of the FE_NO_ at the end of the expiration phase and the expiration flow rate is well reproduced by the model (solid line). In Figure 4B, this link between the expiration flow rate and the value of the FE_NO_ at the end of the respiratory cycle, determined on a single healthy patient by Silkoff et al. (1997), is also presented (dashed line).

A third characteristic of the pulmonary NO transport is related to the link between the duration of a breath-hold phase and the time evolution of the FE_NO_ during the expiration phase of a respiratory cycle (Gabbay et al., 1998). When a sufficient long breath-hold phase is realized, the accumulation of the NO in the airways leads to the presence of a maximum in the plot of the FE_NO_ vs. time during the expiration phase: the FE_NO_ quickly rises during a few seconds, until reaching a maximal value. This maximal value increases if the duration of the breath-hold phase increases. Then, the FE_NO_ rapidly decreases, until reaching a local minimal value. Finally, during the rest of the expiration phase, the FE_NO_ slowly increases (Gabbay et al., 1998). In Figure 4C, the time evolution of the FE_NO_ during the expiration phase of a respiratory cycle, calculated with the model for healthy lungs (using the parameters values given in Tables 1, 2), is presented, for different values of the breath-hold duration time. It is observed that the expected link between the time evolution of the FE_NO_ and the duration of the breath-hold phase is well reproduced by the model.

Several authors also point out different characteristics of the gaseous NO concentration profile in the lungs during a classical respiratory cycle. In the 2–3 last generations, the gaseous NO concentration remains almost homogeneous and constant during the entire cycle, at a value of approximately 2–3 ppb (Pietropaoli et al., 1999; Shin et al., 2001; Van Muylem et al., 2003). This physiological value is the result of a quasi-steady balance between NO production and consumption in these generations. At the end of the respiration phase, the gaseous NO concentration profile exhibits a maximum approximately at the boundary between the alveolar part and the bronchial part of the lungs (i.e., approximately in generation 17). At the end of the expiration phase, the gaseous NO concentration is almost constant in generations 0–7 (value of approximately 15 ppb). Then, it significantly decreases in the so-called intermediate zone of the lungs (generations 8–17), until reaching a value of approximately 2–3 ppb in the last generations. It is mentioned in the next section that, starting from generation 16–17, diffusion begins to be the dominant mechanism of gaseous mass transport. As it appears that the gaseous NO concentration gradient in generations 18 to 20 is pointing toward the mouth during the entire respiration cycle, a diffusion flux of NO toward the end of the lungs is permanently taking place in these generations during the cycle (even during expiration). This phenomenon is commonly called back-diffusion (Van Muylem et al., 2003). In Figure 4D, the gaseous NO concentration profile in the lungs, calculated with our model for healthy lungs, is presented at the end of the inspiration phase and at the end expiration phase of a classical respiratory cycle. Data presented in Tables 1, 2 have been used. It shows that the calculated gaseous NO concentration profiles exhibit the characteristics mentioned above.

**Figure 5:**
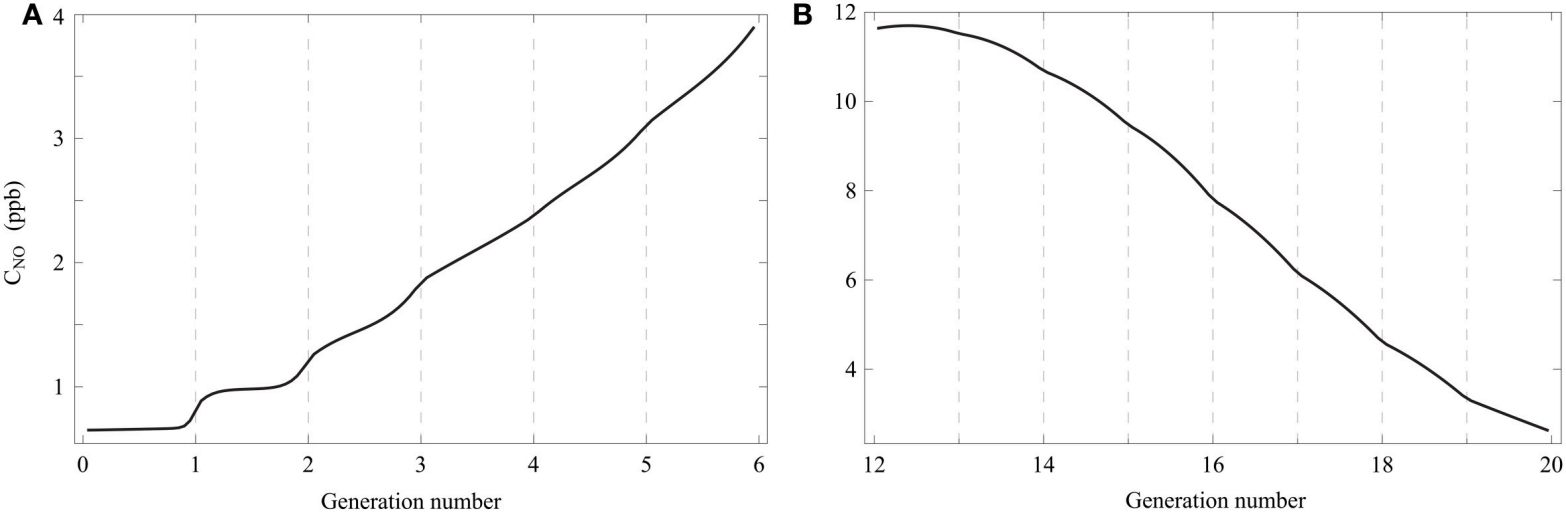
**Gaseous NO concentration profile in healthy lungs, calculated with the model, at the end of a 2 s breath hold phase within a respiratory cycle**. Except for *t*^bh^, all other parameters values are the ones given in Tables 1, 2. **(A)** Generations 0–5. **(B)** Generations 12–19.

The use of the model to simulate a respiratory cycle with a short breath hold phase (2 s) allows highlighting an interesting feature of gas diffusion in the lungs. In Figures 5A,B, the gaseous NO concentration profile in healthy lungs, calculated with our model, is presented at the end of a 2 s breath hold phase within a respiratory cycle. Except for *t*^bh^, all other parameters values are the ones given in Tables 1, 2. It can be observed in Figure 5B that, in the last generations of the lungs, the gaseous NO concentration profile is almost at quasi steady-state at the end of the breath hold phase. Indeed, diffusion is the only mechanism of gaseous NO mass transport during a breath hold phase and it can be observed in Figure 5B that, in each of these generations, the NO concentration profile is almost linear. This can be explained by the fact that, in the last generations, a characteristic time of the axial transport of the gaseous NO by diffusion ($L_{i}^{2}/D_{\text{NO,air}}$) is small compared to the breath hold duration. For instance, in generation 15, $L_{i}^{2}/D_{\text{NO,air}}$ is approximately equal to 0.2 s. On the other hand, it can be observed in Figure 5A that, in the first generations of the lungs, the gaseous NO concentration profile is far from being at quasi steady-state. Indeed, it can be observed in Figure 5A that, in each of these generations, the NO concentration profile is far from being linear. The concentration gradients remain located near the interface between these generations. This can be explained by the fact that, in the first generations, $L_{i}^{2}/D_{\text{NO,air}}$ is large compared to the breath hold duration. For instance, in generation 3, $L_{i}^{2}/D_{\text{NO,air}}$ is approximately equal to 16 s.

**Figure 6:**
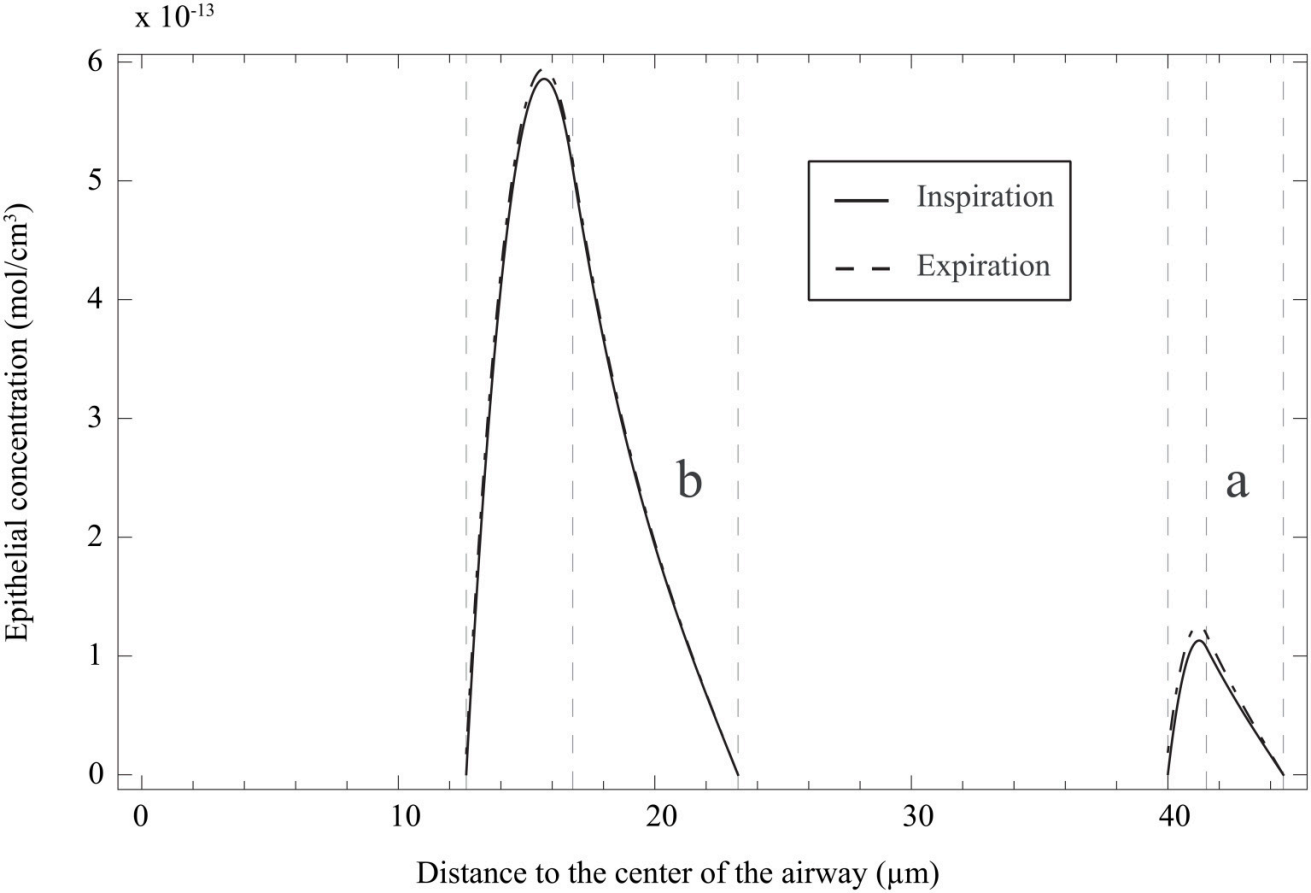
**(a)** NO concentration profile in the layers composing an airway wall, at the position ζ = 1 in generation 12 of healthy lungs, at the end of the inspiration and the expiration phases of a classical respiratory cycle. Data presented in Tables 1, 2 have been used. The concentration is here reported as a function of the distance to the center of the airway. **(b)** NO concentration profile in the layers composing an airway wall, at the position ζ = 1 in generation 12 of unhealthy lungs (cumulative BC up to generation 15, with β = 0.9, no mucus) at the end of the inspiration and the expiration phases of a classical respiratory cycle. Data presented in Tables 1, 2 have been used. The NO concentration is here reported as a function of the distance to the center of the airway.

It is also interesting to use the model [including the solutions of Equation (25) with boundary conditions 26 and 27, given in **Appendix A**] to analyze the NO concentration profile in the layers composing an airway wall, at different moment of a respiratory cycle. In Figure 6A, this profile is presented, at the position ζ = 1 in generation 12 of healthy lungs, at the end of the inspiration and the expiration phases of a classical respiratory cycle. Data presented in Tables 1, 2 have been used. As it might have been expected, the NO concentration reaches a maximum in the epithelial layer. It means that the NO produced in the epithelial layer is partially transferred to the blood and partially to the gas in the lumen.

### 3.2. Dimensionless numbers

Three important dimensionless numbers appear in the model: two Hatta numbers [see Equation (30)] and the Péclet number [see Equation (36)].

Two Hatta numbers (Ha) are defined for each generation. A Ha number compares a characteristic time of NO consumption in a tissue composing an airway wall (epithelial layer or muscle layer) to a characteristic time of transport by diffusion in this tissue. The two Ha numbers introduced in this work are proportional to the epithelial layer thickness and to the muscles layer thickness, respectively. In healthy lungs, these thicknesses are the same in each generation, and they experience variations during a respiratory cycle. Using the model and the data given in Tables 1, 2, it can be calculated that these variations are relatively small and that Ha_*i*_ is around 0.7 and Hã_*i*_ around 0.3 at any time during a respiratory cycle and for each generation. Hence, the NO consumption in the tissues has a moderate influence on the NO concentration profile in these tissues. More precisely, using the model (including the solutions of Equation (25) with boundary conditions 26 and 27, given in **Appendix A**), it can be evaluated that approximately 10 % of the NO produced in the epithelial layer of an airway is consumed in the airway wall. On the other hand, BC can induce an important relative increase of the thicknesses of the epithelial and muscles layers. Using the model and the data given in Tables 1, 2, it can be calculated that BC can lead to values of Ha_*i*_ up to 1 and to values of Hã_*i*_ up to 0.5. Hence, when BC occurs in a generation, the NO consumption in the tissues in this generation has a stronger influence on the NO concentration profile in these tissues than without BC. More precisely, using the model, it can be evaluated that up to approximately 25% of the NO produced in the epithelial layer of an airway can be consumed in the airway wall, if BC has occurred in the airway. This short analysis shows that BC can have a significant influence on the mechanisms of NO transport in the tissues composing an airway wall.

A Péclet number (Pe) is defined for each generation. It compares a longitudinal convective characteristic time to a longitudinal diffusion characteristic time. As shown in Table 1, the value of Pe strongly depends on the generation number. As seen in Equation (36), Pe is proportional to the length of the generation and inversely proportional to the flow cross-sectional area in the generation (when the lungs are at rest). Thus, Pe decreases when the generation number increases. In the long and large first generations, it appears that Pe is far larger than 1. Hence, the NO transport in the first generations is controlled by convection. On the other hand, Pe becomes smaller than 1 in the last generations, indicating that the NO transport in these generations is controlled by diffusion.

### 3.3. Unhealthy lungs

In this section, the model is used to discuss some features of the NO transport in unhealthy lungs.

Several authors showed that the FE_NO, 50_ is modulated by the level of BC (de Gouw et al., 1998; Ho et al., 2000). For instance, experimental data collected by Verbanck et al. (2008) show that the FE_NO, 50_ can decrease if a BC is medically induced in healthy patients.

To analyze with our model the NO transport in unhealthy lungs in which BC has occurred (and with the possible presence of a mucus layer coating the walls of the airways in some generations), several parameters are introduced in order to compare these unhealthy lungs and the corresponding healthy lungs experiencing a same respiratory cycle (i.e., the parameters listed in Tables 1, 2 take the same values for the healthy and the unhealthy lungs). Each of these parameters is defined as the relative difference between a property of the unhealthy lungs and the same property for the healthy lungs. These properties are the FE_NO, 50_ (relative difference written Δ*F*E_NO, 50_), the average value of the NO exchange flux density between the epithelial layer and the lumen of the airways in the last generation impacted by the BC and at the end of the expiration phase (relative difference written Δ*J*), and the total flux of NO from the epithelial layer to the lumen of the airways in the last generation impacted by the BC and at the end of the expiration phase (relative difference written Δ*Flux*).

**Figure 7:**
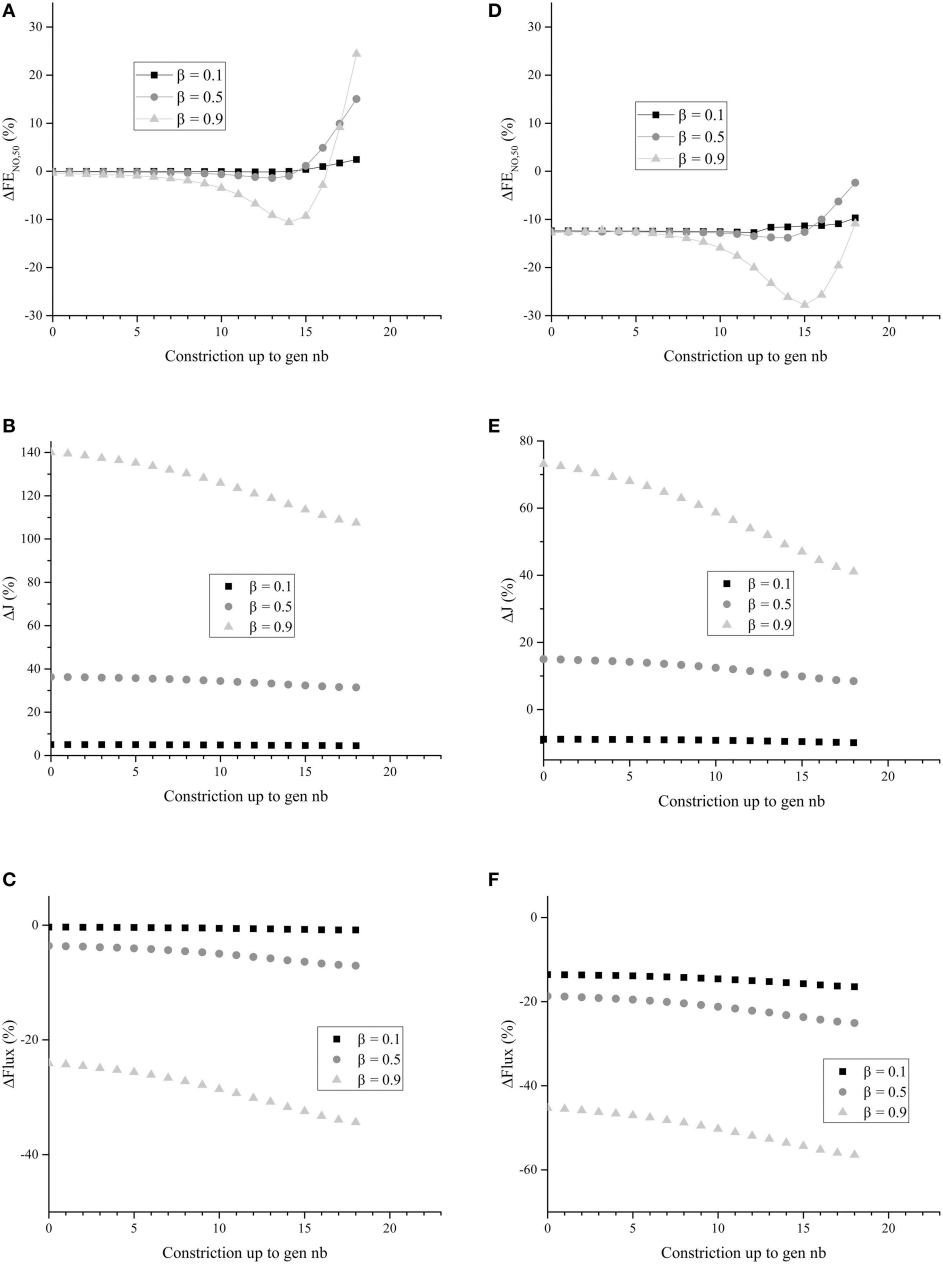
**(A)** Δ*F*E_NO, 50_ for unhealthy lungs in which BC occurs from generation zero up to a given generation, for different values of β. **(B)** Δ*J* for unhealthy lungs in which BC occurs from generation zero up to a given generation, for different values of β. **(C)** Δ*Flux* for unhealthy lungs in which BC occurs from generation zero up to a given generation, for different values of β. **(D)** Δ*F*E_NO, 50_ for unhealthy lungs in which BC occurs from generation zero up to a given generation, for different values of β. Generations 0–18 are coated with a mucus layer of 5 μ*m* thick (before constriction). **(E)** Δ*J* for unhealthy lungs in which BC occurs from generation zero up to a given generation, for different values of β. Generations 0–18 are coated with a mucus layer of 5 μ*m* thick (before constriction). **(F)** Δ*Flux* for unhealthy lungs in which BC occurs from generation zero up to a given generation, for different values of β. Generations 0 to 18 are coated with a mucus layer of 5 μ*m* thick (before constriction). The parameters values given in Tables 1, 2 were used to generate these figures.

In Figures 7A–C Δ*F*E_NO, 50_, Δ*J* and Δ*Flux* are presented for unhealthy lungs in which BC occurred from generation 2 up to a given generation (this is called cumulative BC; it is assumed that BC cannot occur in the first two generations), for different values of β, and without any mucus layer. Homogeneous BC is assumed, i.e., all the generations affected by BC are characterized by the same value of β. The parameters values given in Tables 1, 2 were used to generate these figures.

It can be observed in Figure 7B that Δ*J* is positive, whatever the extent of the BC and the value of β. Moreover, Δ*J* increases if β increases. If BC occurs in an airway, it does not modify the volume of the epithelium and hence the amount of NO produced per unit time in the airway. On the other hand, if BC occurs in an airway, it has two opposite effects on the mechanisms of NO transport in the airway wall. First, as mentioned previously, the Hatta numbers of the concerned generation are increased by BC. It tends to decrease the NO concentration gradient at the interface between the epithelial layer and the lumen. Second, the increase of the muscles and epithelial layers thicknesses leads to an increase of the residence time of the produced NO in the airway wall. Therefore, BC tends to increase the NO concentration in the layers composing the airway wall (see Figure 6B). Moreover, as diffusion is the mechanism of mass transport in the airway wall, a characteristic time of this mass transport is proportional to the square of the wall thickness. Therefore, BC tends to increase the ratio of the maximal NO concentration in the airway wall to the wall thickness, and thus the NO concentration gradient at the interface between the epithelial layer and the lumen. This effect can also be understood by noting that, when solving an 1D diffusion equation in a slab, with a constant volumetric production term and concentrations equal to zero at the slab extremities, it is calculated that the maximal concentration in the slab is proportional to the square of the slab thickness and that the concentration gradient at the slab extremities are proportional to the slab thickness. As Δ*J* is calculated as being positive, it appears that the first of these effects is overwhelmed by the second one (due to the low values of the Hatta numbers). The effect of BC on the NO concentration profile in the layers composing an airway wall can be be observed in Figure 6B. This figure presents this profile, at the position ζ = 1 in generation 12 of unhealthy lungs (cumulative BC up to generation 15, with β = 0.9, no mucus) at the end of the inspiration and the expiration phases of a classical respiratory cycle. In Figure 6B, these profiles are compared with the corresponding ones in healthy lungs. Data presented in Tables 1, 2 have been used to generation these profiles.

It can be observed in Figure 7C that Δ*Flux* is negative, whatever the extent of the BC and the value of β. It means that, in the generations impacted by the BC, the decrease of the exchange surface between the epithelium and the lumen does more than compensate the above mentioned increase of the NO exchange flux density between the epithelial layer and the lumen of the airways. This might have been expected when looking at the results presented in Figure 7B. For instance, for β = 0.9, it is observed in Figure 7B that the NO exchange flux density between the epithelial layer and the lumen of the airways impacted by the BC is increased by a factor between 2 and 2.5. On the other hand, according the the definition of β, when BC occurs in an airway with β = 0.9, the exchange surface between the epithelium and the lumen is decreased by a factor $\sqrt{1-\beta }=0.3$.

It can be observed in Figure 7A that, as expected, cumulative BC influences the FE_NO, 50_. When cumulative BC is limited to the so-called proximal zone of the lungs (generations 0 to 7), Δ*F*E_NO, 50_ is close to zero; the FE_NO, 50_ is almost unaffected by the BC. It can be explained by the fact that the total amount of NO produced in these generations is small, when compared to the total NO production in the lungs. Indeed, the total volume of the epithelial layers in the generations 0–7 is approximately 0.2% of the total volume of the epithelial layers in the lungs. As a consequence, when it occurs only in the proximal zone of the lungs, BC almost does not affect the FE_NO, 50_. When cumulative BC extends up to the so-called central and distal zones of the lungs (generations 8–16), it results in a decrease of the FE_NO, 50_ (negative values of Δ*F*E_NO, 50_). This is coherent with the results of Verbanck et al. (2008). It can be explained by the fact that a significant part of the total NO production takes place in generations 8–16. As a consequence, when BC occurs in these generations, it can significantly decrease the FE_NO, 50_, due to the fact that, as mentioned previously BC leads to a decrease of the total flux of NO from the epithelial layer to the lumen of the airways in the generations impacted by the BC. Finally, when cumulative BC extends beyond generation 16, it appears that the FE_NO, 50_ is increased (positive values of Δ*F*E_NO, 50_). This can be attributed to the fact that, when BC extends beyond generation 16, it blocks the back-diffusion of the NO, and thus promotes the transport of the produced NO toward the mouth. This more than compensates the decrease of the total flux of NO from the epithelial layer to the lumen of the airways in the generations impacted by the BC.

In Figures 7D–F, Δ*F*E_NO, 50_, Δ*J* and Δ*Flux* are presented for unhealthy lungs in which BC occurred from generation 0 up to a given generation, for different values of β, and with a mucus layer of 5 μm thick (before BC) in the airways up to generation 18. Homogeneous BC is assumed again. The parameters values given in Tables 1, 2 were used to generate these figures. It is observed that the presence of mucus layers leads to a decrease of Δ*F*E_NO, 50_, Δ*J* and Δ*Flux*, when compared to the results presented in Figures 7A–C. It seems logical, as the mucus acts as a physical barrier between the site of the NO production (the epithelium) and the lumen. It is interesting to note that, depending on the value of β and of the thickness of the mucus layer coating the airways, Δ*J* can be positive or negative (see Figure 7E).

In conclusion, the use of our model shows that the relation between BC and FE_NO, 50_ is complex. It indicates that BC might lead to an increase or to a decrease of the FE_NO, 50_, depending on the extent of the BC and on the possible presence of mucus. This should be confirmed experimentally and might provide an interesting way to characterize the extent of BC in unhealthy patients.

### 3.4. Model assumptions analysis

As mentioned previously, in order to determine Equations (29) and (31), allowing to express *J*_air, *i*_ in the presence or in the absence of mucus layers in generation i, two main assumptions are made, regarding the transport of NO in the layers composing the wall of the airways in this generation:

- the NO transport equations in the layers can be written as 1D transport equations in a cartesian coordinate system;
- the time derivative of the NO concentration in the layers can be neglected in these equations, i.e., the NO transport in the layers can be considered as being quasi-steady.

The first assumption gives accurate results for a given generation if the radius of the lumen of the airways in this generation is at least one order of magnitude larger than the thicknesses of the layers composing the airways walls. According to the data mentioned in Tables 1, 2, these layers thicknesses are less than 10% of the lumen radius, even in generation 18 (the one exhibiting the smallest lumen diameter among those for which the layered representation of the wall of the airways is considered), in the case of healthy lungs.

The second assumption gives accurate results for a given respiratory cycle if the characteristic times of the NO transport by diffusion in the layers composing an airway wall (*t*_*D*_) are at least one order of magnitude smaller than the inspiration and expiration times of the cycle. If the order of magnitude of the layers thicknesses is 10 μm and if the diffusion coefficient of NO in these layers is approximately 10^−9^ m^2^ s^−1^, *t*_*D*_ is close to 0.1 s^−1^. For a classical respiratory cycle (see Table 2), *t*_*D*_ is thus indeed at least an order of magnitude smaller than the inspiration and expiration times.

In order to check more precisely if these two assumptions are appropriate, we have simulated numerically the NO transport in the epithelial and muscles layers of an airway in a given generation in lungs at rest, in response to a sudden increase (from 0 to 5 ppb) of the NO concentration in the lumen. The airway wall was considered as being a hollow cylinder (i.e., the transport equations in the layers composing the airway wall were written in cylindrical coordinates, assuming axisymmetry) and the time derivative in the transport equations were considered. Data given in Table 2 have been used. Results have shown that, whatever the considered generation and even in the case of a strong BC (β = 0.9), after a transient period (duration of approximately 0.2 s), the calculated total flux of NO from the epithelial layer to the lumen of the airway is close (relative difference less than 10%) to the one obtained when considering the two assumptions mentioned earlier.

## 4. Conclusion

In this work, a new model of the NO transport in the human lungs is presented and used. It belongs to the family of the so-called morphological models and it is based on the morphometric model of Weibel (Weibel, 1963). When compared to previous models, its main new features are the layered, time-dependent, representation of the wall of the airways and the possibility to simulate the influence of bronchoconstriction and of the presence of mucus on the NO transport in lungs. Furthermore, the model is developed in a dimensionless form. It brings out typical dimensionless numbers such as Péclet and Hatta numbers.

The model is based on a geometrical description of the lungs, at rest and during a respiratory cycle, coupled with transport equations, written in the layers composing an airway wall and in the lumen of the airways.

It has been checked that the model is able to reproduce experimental information available in the literature. The model has been used to discuss some features of the NO transport in healthy and unhealthy lungs. The simulation results were analyzed, in order to give new insights into the NO transport in the human lungs, especially when BC has occurred in the lungs. For instance, it has been shown that BC can have a significant influence on the NO transport in the tissues composing an airway wall. BC increases the NO exchange flux density between the epithelial layer and the lumen of an airway (due to the increase of the epithelial and muscles layers thicknesses) but decreases the total flux of NO from the epithelial layer to the lumen of an airway (due to the decrease of the exchange surface). It has also been shown that the relation between BC and FE_NO, 50_ is complex. It indicates that BC might lead to an increase or to a decrease of the FE_NO, 50_, depending on the extent of the BC and on the possible presence of mucus. This should be confirmed experimentally and might provide an interesting way to characterize the extent of BC in unhealthy patients.

## Author contributions

CK, BH, and AV designed the main characteristics of this article. CK and BH constructed the new proposed model based on the one of AV. CK and BH wrote the article.

### Conflict of interest statement

The authors declare that the research was conducted in the absence of any commercial or financial relationships that could be construed as a potential conflict of interest.

## Appendix A

The solutions of Equation (25) with boundary conditions 26 and 27 are:

(A1) $$\begin{array}{l} C_{E,i}\left(x\right)=\frac{Pr}{k}-\frac{e^{{\text{Ha}}_{i}\;+\;{\text{Hã}}_{i}\;+\;x\frac{k}{D_{\text{NO},t}}}\left(\left(-2\;+\;e^{{\text{Hã}}_{i}}\left(1\;+\;{\text{Mu}}_{i}\right)\;+\;e^{2{\text{Ha}}_{i}\;+\;{\text{Hã}}_{i}}\left(1\;+\;{\text{Mu}}_{i}\right)\right)Pr\;+\;2k\lambda_{\text{air},i}C_{i}\left(t,z\right)\right)}{2k\left(-1\;+\;{\text{Mu}}_{i}\;+\;e^{2\left({\text{Ha}}_{i}\;+\;{\text{Hã}}_{i}\right)}\left(1\;+\;{\text{Mu}}_{i}\right)\right)}\\ \;+\;\frac{e^{-{\text{Ha}}_{i}\;+\;x\frac{k}{D_{\text{NO},t}}}\left(\left(1-2e^{2{\text{Ha}}_{i}\;+\;{\text{Hã}}_{i}}-e^{2{\text{Ha}}_{i}}\left(-1\;+\;{\text{Mu}}_{i}\right)-{\text{Mu}}_{i}\right)Pr\;+\;2e^{2{\text{Ha}}_{i}\;+\;{\text{Hã}}_{i}}k\lambda_{\text{air},i}C_{i}\left(t,z\right)\right)}{2k\left(-1\;+\;{\text{Mu}}_{i}\;+\;e^{2\left({\text{Ha}}_{i}\;+\;{\text{Hã}}_{i}\right)}\left(1\;+\;{\text{Mu}}_{i}\right)\right)} \end{array}$$

(A2) $$\begin{array}{l} C_{M,i}\left(x\right)=-\frac{e^{{\text{Ha}}_{i}-x\frac{k}{D_{\text{NO},t}}}\left(\left(-1\;+\;e^{{\text{Hã}}_{i}}\right)\left(-1\;+\;{\text{Mu}}_{i}\;+\;e^{{\text{Hã}}_{i}}\left(1\;+\;{\text{Mu}}_{i}\right)\right)Pr\;+\;2e^{{\text{Hã}}_{i}}k\lambda_{\text{air},i}C_{i}\left(t,z\right)\right)}{2k\left(-1\;+\;{\text{Mu}}_{i}\;+\;e^{2\left({\text{Ha}}_{i}\;+\;{\text{Hã}}_{i}\right)}\left(1\;+\;{\text{Mu}}_{i}\right)\right)}\\ \;+\;\frac{e^{{\text{Ha}}_{i}\;+\;x\frac{k}{D_{\text{NO},t}}}\left(\left(-1\;+\;e^{{\text{Hã}}_{i}}\right)\left(-1\;+\;{\text{Mu}}_{i}\;+\;e^{{\text{Hã}}_{i}}\left(1\;+\;{\text{Mu}}_{i}\right)\right)Pr\;+\;2e^{{\text{Hã}}_{i}}k\lambda_{\text{air},i}C_{i}\left(t,z\right)\right)}{2k\left(-1\;+\;{\text{Mu}}_{i}\;+\;e^{2\left({\text{Ha}}_{i}\;+\;{\text{Hã}}_{i}\right)}\left(1\;+\;{\text{Mu}}_{i}\right)\right)} \end{array}$$

(A3) $$\begin{array}{l} C_{\mu ,i}(x)=\lambda_{\text{air},i}C_{i}(t,z)\;+\;\frac{({\text{Ha}}_{i}\;+\;{\text{Hã}}_{i}\;+\;{\text{Mu}}_{\text{i}})\left(\left(-1\;+\;e^{{\text{Hã}}_{i}}\right)\left(1\;+\;e^{2\;{\text{Ha}}_{i}\;+\;{\text{Hã}}_{i}}\right)Pr-\left(1\;+\;e^{2\;({\text{Ha}}_{i}\;+\;{\text{Hã}}_{i})}\right)k\lambda_{\text{air},i}C_{i}(t,z)\right)}{k\left(-1\;+\;{\text{Mu}}_{i}\;+\;e^{2\;({\text{Ha}}_{i}\;+\;{\text{Mu}}_{i})}(1\;+\;{\text{Mu}}_{i})\right)}\\ \;+\;\frac{x\frac{k}{D_{\text{NO},t}}\left(\left(1-e^{{\text{Hã}}_{i}}\right)\left(-1\;+\;e^{2\;{\text{Ha}}_{i}\;+\;{\text{Hã}}_{i}}\right)Pr\;+\;\left(1\;+\;e^{2\;({\text{Ha}}_{i}\;+\;{\text{Hã}}_{i})}\right)k\lambda_{\text{air},i}C_{i}(t,z)\right)}{k\left(-1\;+\;{\text{Mu}}_{i}\;+\;e^{2\;({\text{Ha}}_{i}\;+\;{\text{Mu}}_{i})}(1\;+\;{\text{Mu}}_{i})\right)} \end{array}$$

## Notations

### Roman symbols

BC: Bronchoconstriction
*C*: Concentration, ml/cm^3^ or mol/cm^3^
*d*: Inner diameter of the alveoli, μm
*D*: Diffusion coefficient, cm^2^/s
Dil: Ratio between the total inspired air volume and the initial gas volume in the lungs, -
*f*: Ratio of the volume of gas in a generation to the total volume of gas in the lungs, when they are at rest, -
Ha: Hatta number, -
Hã: Hatta number, -
Hb: Hemoglobin
*J*: NO exchange flux density, ml/(cm^2^.s)
*k*: kinetic constant, *s*^−1^
*L*: Length of the airways in a generation, cm
Mu: Dimensionless number appearing in Equation (29), -
NO: Nitric oxide
Pe: Péclet number, -
*P*: Total alveolar NO production rate, ml/s
*Pr*: Volumetric NO production rate in an epithelial layer, mol/(cm^3^.s)
*Q*: Gas flow rate, ml/s
*R*: Distance between the center of an airway and the surface of a layer, cm
*S*: Exchange surface, cm^2^
*t*: Time, s
*U*: Total alveolar consumption rate, cm^3^/s
*V*: Total gas volume in the lungs, ml
*x*: Coordinate, cm
*z*: Axial coordinate in an airway, cm

### Greek symbols

δ: Tissue thickness, cm
ζ: Dimensionless axial coordinate in an airway, -
λ: Equilibrium constant, mol/cm^3^
γ: Correcting coefficient, cm^3^/mol
θ: Dimensionless time, -
Ω: Total cross-sectional area in a generation, cm^2^
Ω′: Total flow cross-sectional area in a generation, cm^2^

### Subscripts

0: At rest
air: Airway
alv: Alveolus
*D*: Diffusion
*E*: Epithelium
*i*: Generation i
*L*: Lumen
*M*: Muscle
μ: Mucus
max: Maximal value
NO: Nitric oxide
*t*: Tissue
tot: Total (sum over the 24 generations)

### Superscripts

bh: Breath-hold
ex: Expiration
in: Inspiration
